# Redox-state-dependent CO binding in MoFe nitrogenase: insights from EPR, IR, and QM/MM analysis

**DOI:** 10.1039/d6sc05535d

**Published:** 2026-10-07

**Authors:** Atanu Rana, Justin P. Joyce, Zachary Mathe, Casey Van Stappen, Kushal Sengupta, Christian Lorent, Ingo Zebger, Laure Decamps, Serena DeBeer

**Affiliations:** a Max Planck Institute for Chemical Energy Conversion Stiftstrasse 34-36 45470 Mülheim an der Ruhr Germany serena.debeer@cec.mpg.de; b Institut für Chemie, PC14, Physikalische und Biophysikalische Chemie, Technische Universität Berlin Strasse des 17. Juni 135 10623 Berlin Germany

## Abstract

Nitrogenase drives biological nitrogen fixation through an eight-step cycle, in which “*n*” electrons and protons are accumulated across the E_*n*_ catalytic states. Carbon monoxide (CO), a noncompetitive inhibitor for Mo nitrogenase but a substrate for V- or Fe-only nitrogenases, has been widely used as a spectroscopic probe of nitrogenase's active site. Infrared (IR) spectroscopy reports on CO binding mode and coordination environments, while electron paramagnetic resonance (EPR) spectroscopy characterizes CO-bound states *via* their non-integer spin states. Crystal structures reveal a µ_2_-bridging mode formed by substitution of a “belt” sulfide, suggesting that sulfide lability may be mechanistically relevant. However, the redox state at which CO binds and the assignment of the resulting non-integer spin states remain unresolved, obscuring their relationship to the N_2_ reduction cycle. Likewise, the mechanism and relevance of sulfide substitution remain unclear. Here, we combine static and stopped-flow IR, EPR spectroscopy, photolysis, and quantum mechanical/molecular mechanical (QM/MM) calculations, supported by non-negative matrix factorization (NMF), to investigate CO binding under varying electron flux, varying CO pressure, and H_2_ pressure. We find that CO binds predominantly at the E_1_ state, whereas EPR-active species correspond to CO-bound E_2_ states. H_2_S is identified as the preferred leaving group, and the absence of hydrides supports a stable CO-bound state that is disconnected from the reductive steps of nitrogen fixation.

## Abstract 2

Combined spectroscopic and computational studies elucidate the mechanism of CO binding and inhibition in the early E_*n*_ states of MoFe nitrogenase.

## Graphical abstract

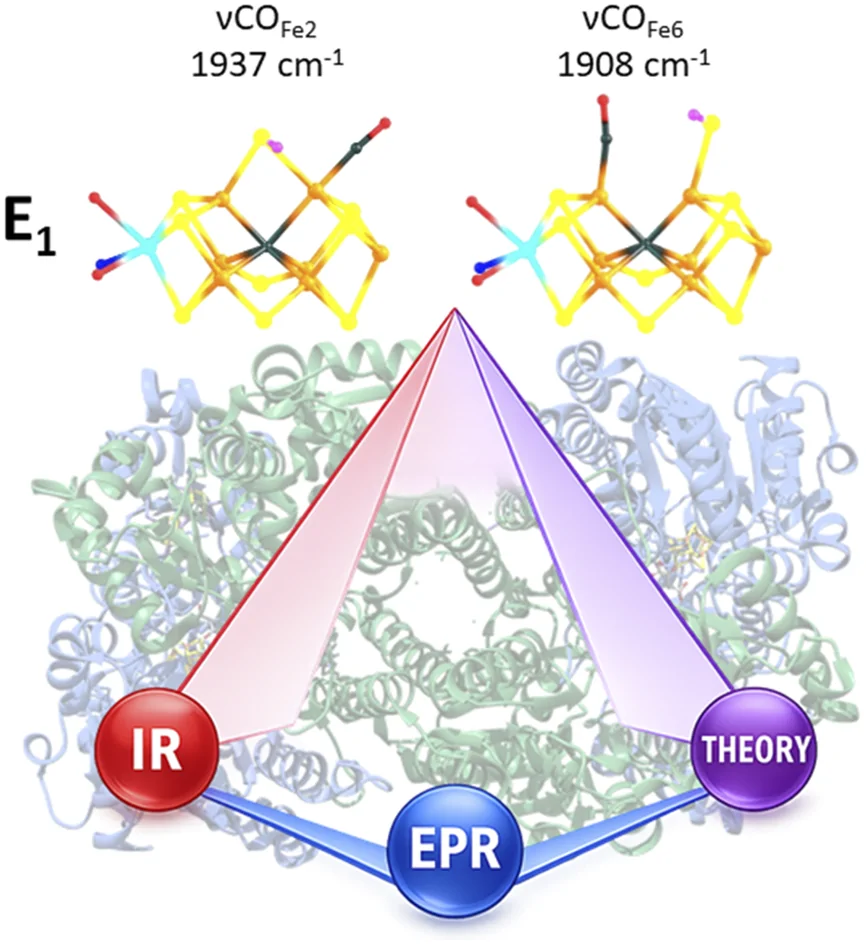

## Introduction

Nitrogenase (N_2_ase) catalyzes the biological conversion of atmospheric dinitrogen (N_2_) into ammonia (NH_3_) under ambient conditions, supporting the biosynthesis of nucleotides and amino acids. Three isoforms of N_2_ases exist, distinguished by the heterometal in their catalytic cofactors: Mo N_2_ase (FeMoco: [MoFe_7_S_9_C]), V N_2_ase (FeVco: [VFe_7_S_8_C(CO_3_)]), and Fe N_2_ase (FeFeco: [Fe_8_S_9_C]).^1–7^ Although all reduce N_2_,^8–12^ they also catalyze the reduction of alternative small molecules.^13–19^ Catalysis requires the reductase Fe protein (FeP), which delivers electrons to the MoFe protein in an ATP-dependent manner through a transient protein–protein complex.^20,21^ Electron and proton accumulation at the active site follows the Lowe–Thorneley (LT) kinetic scheme and stepwise addition of “*n*” electrons and protons at each E_*n*_ state (Fig. 1).^22,23^

**Fig. 1:**
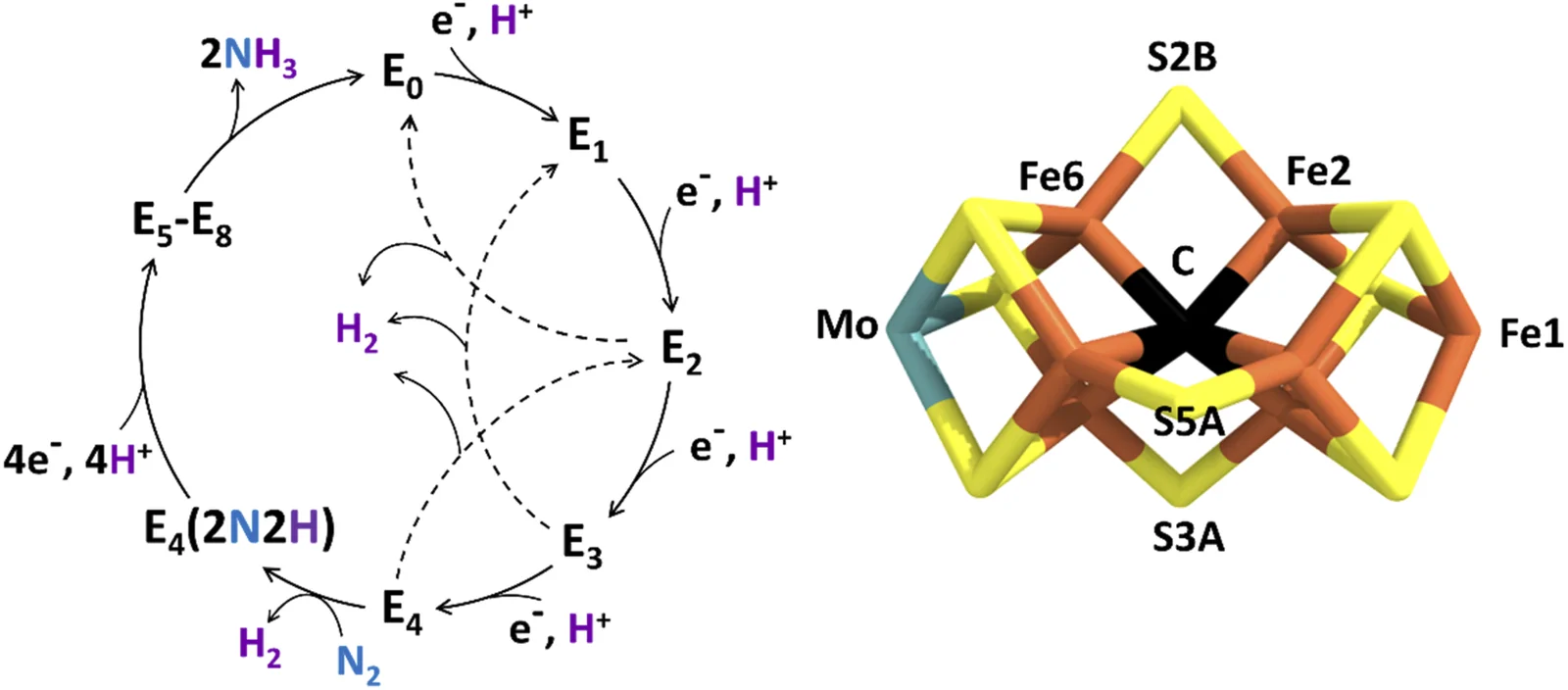
Schematic depiction of the Lowe–Thorneley (LT) cycle, highlighting the suggested intermediates in the Mo nitrogenase-catalyzed reduction of N_2_ (left). The structure of the [MoFe_7_S_9_C]^1−^ cluster of FeMoco in which some key elements are labeled, and the elements are color coded as follows: Fe – orange, S – yellow, Mo – green, and C – black (right).

Carbon monoxide (CO) has long been used as a probe for identifying nitrogenase intermediates. It is a noncompetitive inhibitor of N_2_ reduction by Mo N_2_ase but does not inhibit proton reduction.^24–26^ V N_2_ase, however, catalytically reduces CO to hydrocarbons (C_1_–C_4_),^27^ resembling the Fischer–Tropsch process,^18,28–35^ while Fe N_2_ase predominantly forms methane.^36,37^ Remarkably, some Mo N_2_ase variants with altered residues near FeMoco can also couple and reduce CO to methane and higher hydrocarbons.^38^ These observations underscore shared mechanistic features across nitrogenases and motivate studies into how CO binds and reacts at their active sites.

The observed CO-bound species depend on three factors: timing of measurement relative to turnover, applied CO pressure, and electron flux determined by the [MoFe] : [FeP] ratio. Early EPR studies identified two distinct *S* = 1/2 species: “hiCO” (*g* = [2.17, 2.06, 2.06]) formed under high CO pressure and “loCO” (*g* = [2.09, 1.98, 1.93]) under low CO pressure, with a third *S* = 3/2 species (“hi(5)-CO”) appearing under intermediate flux conditions.^39–41^ Subsequent ^57^Fe and ^13^C ENDOR experiments confirmed both as CO-bound FeMoco species and are interconvertible by adjusting CO pressure^39,42–45^ or *via* low-temperature photolysis.^46^ Additional ^13^C and ^1^H ENDOR studies proposed two terminal CO groups in hiCO and a bridging CO in loCO.^47^ Crystallographic structures later identified Fe2 and Fe6 as binding sites, where a µ_2_CO substitutes their “belt” sulfide, and that Fe6 can further bind a terminal CO group, although with only partial occupancy (Fig. 2).^44,45^ The analogous structures have been also found in CO-bound forms of FeVco, suggesting S2B displacement and µ_2_CO binding may be characteristic across the nitrogenase family.^48,49^

**Fig. 2:**
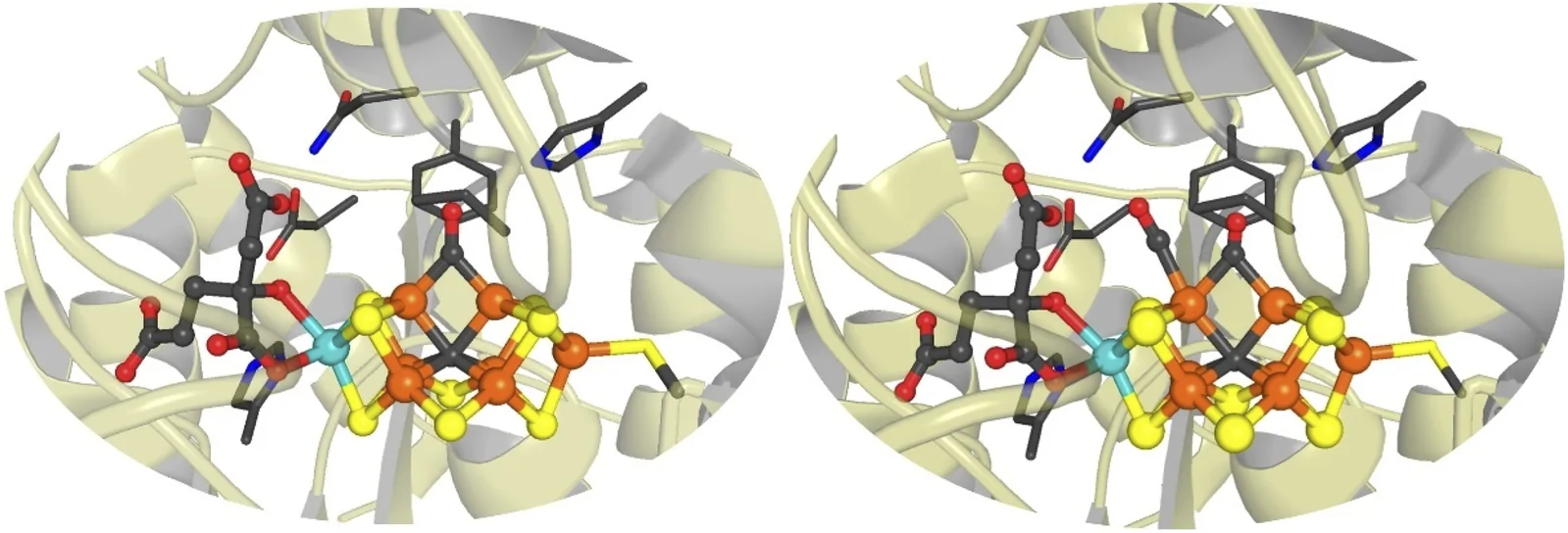
The crystal structure of the single CO-(left, PDB 4TKV^44^) and double CO-bound (right, PDB 7JRF^45^) of FeMoco (depicted as ball and stick model). The residues in the secondary coordination sphere of the CO-bound Fe2 and Fe6 sites are included (Val70, Gln191, His195, Glu380, and Phe381, marked as stick models). The terminal CO of PDB 7JRF is modeled with 50% occupancy.

Time-resolved FT-IR studies detected up to six *ν*(C≡O) bands (1715–1960 cm^−1^), revealing multiple CO-bound FeMoco intermediates.^50–52^ Red-shifted bands (∼1715 cm^−1^) may indicate strongly activated CO rather than formyl species, as supported by recent Fe–S cubane model studies.^53–55^ Despite extensive characterization, correlations between IR and EPR species remain uncertain, as most investigations employ only one spectroscopic technique under specific conditions.^39,40,42,43,50,51,56,57^

CO binding in wild-type MoFe occurs during enzymatic turnover,^47^ but whether it first associates at the E_1_ or E_2_ state remains unresolved. While experimental studies favor EPR active E_2_ as the state at which CO binds,^42,43,47,58^ QM/MM calculations suggest that EPR silent E_1_ could already support CO binding.^59^ Moreover, experimentally, only a fraction of the enzyme contributes to EPR-visible signals, leaving an open question whether EPR-silent species also contain bound CO.^40,60,61^ To answer this, EPR silent species must be decisively correlated with IR data to determine if CO binding first occurs at the E_1_ or E_2_ state.

In addition to the question of which E_*n*_ state first binds CO, the fate of the µ_2_-S2B bridge during turnover still remains unknown. Displacement and complete loss of S2B have now been observed crystallographically and by cryo-EM across multiple nitrogenase structures and in all three isoforms, providing strong evidence that S2B can be displaced to open a coordination site during catalysis.^62,63^ However, our recent SEIRA studies of immobilized MoFe showed that S–H stretches persist upon CO binding, suggesting that complete S2B dissociation is not required for formation of at least some CO-bound states.^64^ Together, these observations point to a dynamic role for S2B, in which its coordination may depend on the catalytic state, substrate, and experimental conditions.

Herein, we combine time-resolved FT-IR, EPR, and photolysis experiments with QM/MM calculations aided by non-negative matrix factorization (NMF) to examine CO-bound state formation in FeMoco as a function of electron flux, reaction time, and CO/H_2_ partial pressures. These integrated results enable correlation between CO-bound EPR and IR species, propose assignments within the Lowe–Thorneley framework, and yield insights into the mechanism of µ_2_-sulfide displacement and CO inhibition.

## Materials and methods

### Materials and protein purification

All reagents were obtained from Sigma-Aldrich (St. Louis, MO) or Fisher Scientific (Fair Lawn, NJ) and used without further purification. MoFe and Fe proteins were isolated from wild-type *Azotobacter vinelandii* as described previously.^65^ All manipulations of the proteins and buffers were performed in a Coy glovebox with a 98% N_2_/2% H_2_ atmosphere. The purities of these proteins were >95% based on sodium dodecyl sulfate polyacrylamide gel electrophoresis analysis with Coomassie staining. Activity of the isolated MoFe and Fe proteins was routinely assessed *via* the acetylene reduction assay following previously published procedures.^13^ For D_2_O saturation, the MoFe and Fe proteins were passed through PD-10 desalting columns (GE Healthcare) equilibrated with D_2_O buffer (composition of the pD 6.9 buffer: 200 mM MOPS, 5 mM MgCl_2_, 150 mM NaCl). The buffer-exchanged proteins were finally concentrated using centrifugal filter units (millipore) and stored anoxically at −80 °C until further use. The concentrations of purified MoFe and Fe proteins were determined by the Lowry assay.

#### Preparation of activating solution

The activation solutions were prepared in a 200 mM MOPS buffer at pD 6.9 with 5 mM MgCl_2_, 150 mM NaCl, 20 mM ATP, and 20 mM sodium dithionite under a 98% N_2_/2% H_2_ atmosphere. For all excess CO (hi-*p*CO) sample preparations, the solution was purged with 100% CO gas (N_2_ : CO = 0 : 1). For sub-stoichiometric CO sample preparations (lo-*p*CO) the solution was purged using a 16% CO in N_2_ gas mixture (N_2_ : CO = 6 : 1). For the H_2_ partial-pressure experiments, the activating solution was purged with an 84 : 16 H_2_ : CO gas mixture and subsequently mixed in a 1 : 1 (v/v) ratio with the previously prepared protein solution inside an atmospheric pressure box.

#### Preparation of turnover EPR samples

All native turnover EPR samples were prepared in a Coy glovebox with a 98% N_2_/2% H_2_ atmosphere. A protein solution was prepared in a 200 mM MOPS buffer at pD 6.9 with 5 mM MgCl_2_, 150 mM NaCl with 100 µM of MoFe protein by the addition of FeP protein with the adequate concentration for the desired electron fluxes (MoFe : Fe 50 : 1, 5 : 1, and 1 : 5) and an activating solution was prepared with 200 mM MOPS buffer at pD 6.9 with 5 mM MgCl_2_, 150 mM NaCl with 20 mM ATP and 20 mM sodium dithionite. The EPR samples were prepared by mixing the protein solution and activating solution 1 : 1 ratio at room temperature in an EPR tube under a 98% N_2_/2% H_2_ atmosphere. The reaction mixtures were then flash-frozen in liquid N_2_ for each time point. Samples were stored in liquid N_2_ until measurement. Because of inherent limitations of the SF-FTIR spectroscopy setup, the ATP regeneration system was excluded and this omission was maintained uniformly across all experimental conditions. To ensure sustained turnover throughout the longer measurement period, a large excess of MgATP was used instead.

#### Electron paramagnetic resonance (EPR) measurements

Continuous-wave (CW) EPR measurements were performed at X-band frequency (∼9.65 GHz) using a Bruker E500 ELEXSYS spectrometer equipped with a Bruker dual-mode cavity (ER4116DM) and an Oxford Instruments helium-flow cryostat (ESR 900). A high-sensitivity Bruker Super-X (ER-049X) bridge with an integrated microwave frequency counter was employed, along with a magnetic field controller (ER032T) that was calibrated using a Bruker NMR field probe (ER035M). All measurements utilized a 7.46 G modulation amplitude with a 100 kHz modulation frequency and a 80.92 ms time constant. The sample temperature was stabilized at 10 K using a He flow cryostat (Oxford Instruments) with 0.5 mW microwave power.

Data handling and spectra simulations were accomplished with the simulation lab (SL) routines eview.SL and esimX.SL written by Dr Eckhard Bill. The routine eview.SL was used for conversion of the Bruker-type binary data into ASCII format, visualization of the spectra, eventual baseline correction for offset and linear slope, and for numerical double integration of the experimental derivative spectra for spin quantitation. Spin quantitation at non-saturating conditions was done with respect to resting FeMoco, which acts as an internal standard for every sample. Temperature differences of reference and target measurements were considered by assuming EPR intensities of *S* = 1/2 systems to be proportional to the inverse temperature and to the square root of power. Differences in spectroscopic *g* values of reference and target spectra were taken into account by using Aasa-Vänngård factors to correct the numerical integrals of the field-sweep spectra to obtain correct, comparable frequency-dependent intensities.^66^

The experimental powder spectra were simulated with the routine esimX.SL using spins *S* = 1/2 with anisotropic *g* values and anisotropic line widths with superpositions of Lorentzian and Gaussian line shapes (pseudo-Voigt lines). The frequency-dependent intensities of subspectra obtained by the powder simulations were used for the determination of relative abundances of species in the samples. The *S* = 3/2 spectrum of the FeMoco unit was also simulated with fictitious spin *S* = 1/2 and effective *g* values. The Boltzmann population of the low-lying |*S* = 3/2, *m*_s_ = ± *S* = 1/2 > resonance doublet was assumed to be 100%, due to the presence of zero-field splitting and the low measuring temperature (12 K). In addition to the nitrogenase spectra plotted here, sharp, isotropic *g* = 2.00 components (<0.5%) were sometimes necessary to include in simulations as the result of an unknown radical contaminant.

#### Stopped-flow (SF) and static FT-IR measurements

SF FT-IR spectra were measured using a Hi-Tech Scientific SF-73 stopped-flow system coupled to a Bruker Vertex 80v FT-IR spectrometer equipped with a mercury cadmium telluride (MCT) detector cooled by liquid N_2_. Spectra were recorded in the double-sided, forward-backward mode, with a resolution of 4 cm^−1^, an aperture setting of 4 mm, and a scan velocity of 140 kHz. The SF FT-IR cell was coupled *via* an air-sealed umbilical to the SF-73 two-syringe rapid mixing tower placed in a glovebox under an N_2_ atmosphere. Protein solution containing 100 µM MoFe mixed with FeP at specified concentrations was mixed in a 1 : 1 ratio with the activating solution. Static FT-IR measurements were conducted using a Bruker Vertex 80v FT-IR spectrometer operating in double-sided, forward-backward mode. The spectrometer was set to a resolution of 2 cm^−1^, an aperture of 3 mm, and a scan velocity of 20 kHz, and it was equipped with a MCT detector cooled by liquid N_2_. Samples were positioned between CaF_2_ windows, separated by a 100 µM Teflon spacer (20 µL sample), and sealed in custom-built brass holders with rubber rings. Both protein and activating solutions were prepared in 200 mM MOPS buffer at pD 6.9 and additionally contained 5 mM MgCl_2_ and 150 mM NaCl. For each shot of SF FT-IR measurement, 120 µL was used.

FT-IR data were processed by subtracting a baseline followed by non-negative matrix factorization (NMF) analysis, a method by which a small number of component spectra are determined that, in varying weights over time, span the time-dependent dataset (*vide infra*). The baseline was determined by asymmetric least-squares (ASLS) using pybaselines;^67,68^ see the SI for raw spectra and fitted baselines. All static data were processed in one NMF analysis, and all SF data were processed in another, due to the different sampling rates in these datasets. NMF analyses were performed using Scikit-learn^69^ with an NNDSVD guess^70^ (*vide infra*). Data for 300–1780 s for every condition were included in the static FT-IR NMF analysis; for low flux (50 : 1) samples, peak trajectories continuing beyond 1800 s were instead determined as peak integrals. Most reaction conditions resulted in reactant consumption and a worsening baseline by this time and a homogeneous time sampling was used to avoid NMF bias towards one condition. In order to plot the NMF weights trajectories for the static and SF datasets together, the trajectories were normalized to the total area of the respective component as well as the renormalized weight of the corresponding “A” component at the last (SF) and first (static) time points (*vide infra*).

#### Photolysis experiments

EPR photolysis measurements employed a LOT-Quantum Design 300 W Xenon arc lamp, powered by a LSN354 power supply. All *in situ* photolysis experiments and EPR measurements were performed at 12 K. The recombination experiments were performed at 77 K. Samples for complementary cryo-IR spectroscopic studies were prepared from the previously measured EPR tubes by thawing the samples and transferring 10 µL of the MoFe protein solution to a custom-made IR sandwich cell, composed of two CaF_2_ widows and a 50 µM Teflon spacer. This procedure was conducted under an anaerobic atmosphere (5% H_2_, 95% N_2_) within 20 min. The IR cell was then mounted into a helium flow cryostat from Oxford instruments incorporated in a Bruker Tensor 27 or an IFS-66 FT-IR spectrometer, equipped with a liquid-nitrogen-cooled MCT detector, allowing spectra acquisition of 5–10 spectra each containing 200 scans with resolution of 2 cm^−1^ at scanner velocity of 20 kHz with an aperture of 3 mm. An LED array (460 nm) was used for illumination performed at 10 K. Broad drifts of the baseline were compensated using a spline function. The OPUS software from Bruker, version 6.5 or higher, was used for spectra acquisition and analysis. It is important to note that all FT-IR photolysis measurements required an additional ∼1800 s for sample preparation (sample cell construction, sample transfer, and cryostat cooling) beyond the sample quenching time in CO turnover described in the text.

### QM/MM calculations

The QM/MM model used for FeMoco has been previously detailed and is based on the 1.0 Å resolve crystal structure of FeMoco's E_0_ state (PDB 3U7Q).^1,71^ The QM/MM calculations were run with the ASH program^72^ that provides a flexible interface to the OpenMM molecular mechanics library^73^ and the ORCA quantum chemistry code (ORCA v5.03).^74,75^ Link atoms and charge-shifting were used to treat the QM/MM boundary.^76^ The geomeTRIC optimization library^77^ was used to optimize the geometries, using HDLC coordinates^78^ of a 959-atom active region defined as all residues within a 10 Å radius from the cofactor's interstitial carbide. The QM-region was selected to include FeMoco's primary and secondary coordination sphere whose residues are Val70, Arg96, Gln191, His195, Cys275, Ser278, Gly356, Gly357, Arg359, Glu380, Phe381, His442 and HOH519.

The r^2^SCAN density functional^79^ was used with the D4 empirical dispersion correction^80,81^ and the ZORA scalar relativistic Hamiltonian^82,83^ whose procedure is benchmarked^84^ for the geometry of iron-sulfur systems. The recontracted ZORA-def2-TZVP^85,86^ relativistic basis set was used for the Fe and S centers and the interstitial carbide. The same basis set was used for the H and CO substrates. The all-electron SARC-ZORA-TZVP^87^ basis set was applied for Mo, while the remaining atoms used the smaller ZORA-def2-SVP basis set. The “SARC/J” keyword was used that pairs the Split-RI-J approximation with a decontracted auxiliary basis set.^85,88^ The vibrational modes were calculated with a partial Hessian matrix, defined as CO and the coordinated Fe-site. The calculated frequencies are not scaled with respect to an empirical constant.^89^

The CO bound isomers for FeMoco's E_1_ and E_2_ states were calculated, considering the terminal or bridging binding modes of CO at the Fe2 and Fe6 sites. The structures each optimized by broken-symmetry density functional theory (BS-DFT). The *M*_s_ = 1 and 2 states were considered for the E_1_ CO bound states while the E_2_ CO states were modeled as *M*_s_ = 1/2. We considered each structure's BS-235, −247, −346 and −147 BS-configurations, referring to the Fe-centers with net β-spin, that have been shown as the most stable for the cofactor.^90^ The Hirshfeld spin population of the metal centers for the selected BS-states are provided in Table S5.^87^ We note that diminished spin densities at the Fe2 and Fe6 sites that can accompany CO binding can require a fourth Fe-site with β-spin. In these instances, we still refer to the structure's initially requested BS-configuration.

The CO binding energies (Δ*E*_CO_) of the [E_*n*_](CO) isomers were calculated by the electronic energy of the CO-bound cluster with respect to their lowest energy E_*n*_ isomer and free CO, optimized at the same level of theory in a continuum solvent^91^ with a dielectric constant of 4.0. The Δ*E*_CO_ for [E_2_](µ_2_CO) was calculated in the presence of the dissociated S2B as H_2_S included in the QM/MM model. The Δ*E*_CO_ for the [E_2_](CO)_2_ isomers was calculated by their electronic energy with respect to [E_2_](µ_2_CO), where the dissociated S2B as H_2_S was removed from the QM/MM model. We note that the dissociated H_2_S had negligible impact on the calculated CO vibrational mode for the [E_2_](µ_2_CO) structures. The formalism for the Δ*E*_CO_ values is shown in eqn (1), where *n* = 1 or 2 and *x* = 1 or 2.

1 Δ*E*_CO_ = *E*_[E_*n*_](CO)_*x*__ − (*E*_[E_*n*_](CO)_*x*−1__ + *E*_CO,CPCM_)

The lowest energy structure for the E_1_ isomer has a protonated S2B bridge with a BS-346 (*M*_s_ = 2) state. The E_2_ isomer's ground state structure has a µ_2_-hydride bridging Fe2 and Fe6, the protonated S2B coordinated terminal to Fe6, with a BS-235 (*M*_s_ = 3/2) state.^64^ The QM-region of the QM/MM optimized geometries of both the E_1_ and E_2_ intermediates are shown in Fig. S19. Entropic contributions to CO binding are not included in the relative electronic energies because the translational and rotational entropy of CO is poorly defined in the protein environment compared with the gas phase.^92–94^

## Results and analysis

Previous studies have established three observations regarding the interaction of CO with Mo N_2_ase: (1) CO binding to FeMoco requires enzymatic turnover,^47^ (2) CO is a potent non-competitive inhibitor for N_2_ reduction (as well as azide) but not H^+^ reduction,^24–26^ and (3) CO binding occurs across a wide range of e^−^ flux conditions (with previously reported conditions spanning from MoFe : FeP = 5 : 1 to 1 : 4).^39,40^ Despite this, the specific redox state(s) of FeMoco that permit CO binding, as well as the structural and electronic nature of the resulting CO-bound cofactors, remain poorly defined. To address these questions, we employed a combination of SF- (and static) FT-IR and freeze-quench perpendicular mode CW X-band EPR. These techniques are complementary in that EPR is half-integer spin sensitive and quantitative while FT-IR is spin-insensitive and not rigorously quantitative. This combined approach therefore enables orthogonal characterization of the time-dependent intermediate species generated under enzymatic turnover conditions both at (a) high and low partial pressures of CO (referred to as hi-*p*CO and lo-*p*CO, and representing excess and sub-stoichiometric concentrations, respectively),^39^ and (b) under low (50 : 1), intermediate (5 : 1), and high (1 : 5) e^−^ flux conditions (referred to as *n* : *m*-flux, [MoFe] : [FeP]).

### Overview of spectroscopic species

Turnover of Mo N_2_ase in the presence of N_2_ and CO results in the accumulation of a multitude of species, several of which have been previously characterized individually by either EPR or IR spectroscopic methods.^7,39,40,42,43,48,49,54,55^ The nomenclature of “hiCO” and “loCO” has been used in many EPR studies (and in some IR studies) starting from the first reports from Orme-Johnson and co-workers;^39,50,52^ we prefix here with “EPR-” to avoid conflating conditions with species. Many of these spectroscopically defined states are operationally named based on the conditions under which they were observed – for example, EPR-hiCO and -loCO reflect species generated under high and low partial pressures of CO, respectively. Additionally, a third *S* = 3/2 species, designated hi(5)-CO and associated with CO-bound FeMoco, has been reported.^41^ Specifically under hi-*p*CO with higher e^−^ flux (1 : 5 and 5 : 1), only negligible amounts of hi(5)-CO were observed in our experiments, and hence is not discussed further in the present study. Herein, we emphasize that care must be taken when correlating spectroscopically defined species across methods, as identical spectroscopic features do not necessarily imply identical chemical structures. To facilitate the following discussion, EPR-detected species are summarized in Table 1 and FT-IR-observed species in Table 2. We broadly summarize all of our EPR and IR data collected across the full set of conditions in these tables, then present subsets of conditions sequentially to rationally build our working assignments and propose a unified mechanistic model of CO binding and inhibition.

**Table 1:** Overview of species defined by EPR spectroscopy; electron fluxes are indicated as MoFe : FeP

State	*g*-values	Reaction conditions	References
E_0_ (resting state)	4.33, 3.63, 2.00	As purified (dithionite-reduced)	95, and this work
EPR-loCO	2.09, 1.97, 1.93	Under lo-*p*CO with high (1 : 5), and intermediate (5 : 1) e^−^ flux	38, 39, 44, and this work
EPR-hiCO	2.17, 2.06, 2.06	Under hi-*p*CO with higher e^−^ flux (>5 : 1)	38, 39 and 44
hi(5)-CO	5.78, 5.15, 2.7	Under hi-*p*CO and high e^−^ flux (1 : 1) turnover conditions	41
EPR-hiCO1	2.17, 2.06, 2.05	Under hi-*p*CO with high (1 : 5), intermediate (5 : 1), and low (50 : 1) e^−^ flux	This work
EPR-hiCO2	2.18, 2.08, 2.05	Under hi-*p*CO with high (1 : 5), intermediate (5 : 1), and low (50 : 1) e^−^ flux	This work
FeP^1+^	2.03, 1.94, 1.86 (Nucleotide free)	Irrespective of conditions	96, and this work

**Table 2:** Overview of species defined by IR spectroscopy. Electron fluxes are indicated as the [MoFe] : [FeP] ratio

NMF component	Vibrational frequencies (cm^−1^)	Reaction conditions	References
—	1880/1906, 1936/1960, 1908	Under hi-*p*CO and high e^−^ flux (1 : 3.4) turnover conditions	47, 50 and 51
—	1904	Under lo-*p*CO and high e^−^ flux (1 : 3.4) turnover conditions	50 and 51
Static-A	∼1908 and ∼1937	Under hi-*p*CO and lo-*p*CO with low (50 : 1), intermediate (5 : 1), and high (1 : 5) e^−^ flux	51, This work
Static-B	1905	Under hi-*p*CO and lo-*p*CO with low (50 : 1), intermediate (5 : 1), and high (1 : 5) e^−^ flux	51, This work
Static-C	1962	Under hi-*p*CO and lo-*p*CO with low (50 : 1), intermediate (5 : 1) and high (1 : 5) e^−^ flux	This work
SF-A	∼1908, ∼1937	Under hi-*p*CO and lo-*p*CO with intermediate (5 : 1) and high (1 : 5) e^−^ flux	51, This work
SF-B	1905	Under hi-*p*CO and lo-*p*CO with intermediate (5 : 1) and high (1 : 5) e^−^ flux	This work

**Photolysis summary**			
—	1972, ∼1715, ∼1680	Under hi-*p*CO and high e^−^ flux (1 : 4) turnover conditions, photolysis vibrations for wild type Mo–N_2_ase	56
—	1972, ∼1715, ∼1680	Under hi-*p*CO with 1 : 5-flux (100 s and 600 s), low (50 : 1), and lo-*p*CO with 1 : 5-flux (3600 s)	This work
Hi-2 [EPR: hi(5)-CO]	1938 and 1874	Under 202 kPa CO pressure with intermediate e^−^ flux (4 : 1)	97

### EPR spectroscopy

The EPR data were collected for a range of e^−^ flux conditions and varied CO partial pressures detailed above. In addition, control experiments were performed in the absence of CO to assess the relative populations of E_*n*_ states in the absence of CO, and to distinguish CO-specific features. Representative EPR spectra collected over time under one condition (lo-*p*CO, 1 : 5-flux) are shown in Fig. 3, along with the corresponding multi-component fits. Spin-Hamiltonian fitting was used to deconvolute the components of the EPR spectra. This approach was preferred over matrix factorization because the EPR subspectra are tightly constrained by the physics captured by the effective spin Hamiltonian, rather than by simple mathematical constraints like non-negativity. The relative populations of the EPR-active and EPR-silent states were determined as a function of electron flux and CO partial pressure and are discussed further in the EPR and IR correlation section.

**Fig. 3:**
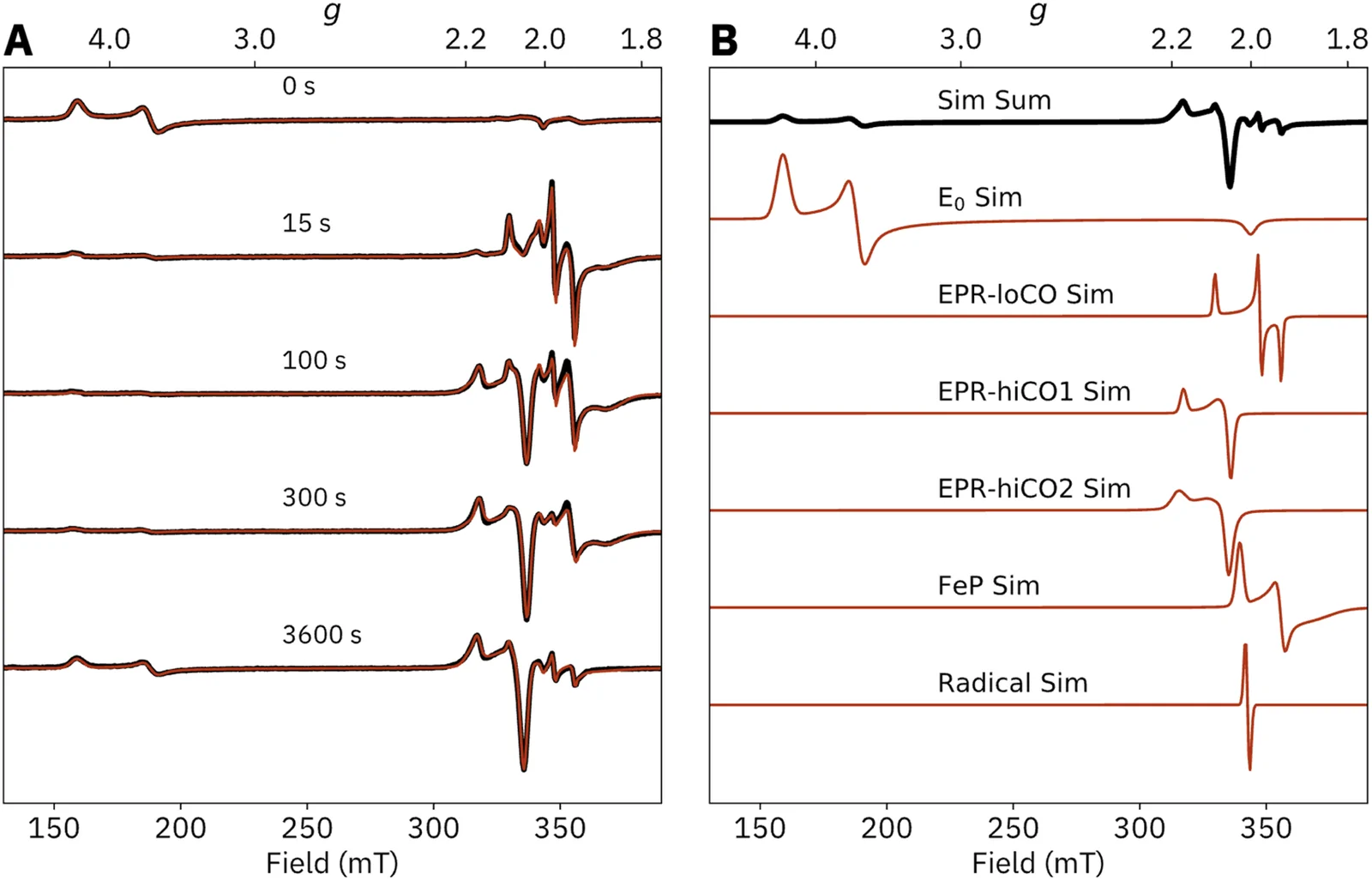
Experimental (black) and simulated (red) EPR spectra for the lo-*p*CO, 1 : 5-flux condition, including (A) spectra at multiple timepoints and the mentioned timepoints are freeze quench timing of the turnover reaction; and (B) the intensity-normalized spectrum of each simulation component for the 3600 s data point. The relative accumulations of the four EPR-active components (E_0_, EPR-hiCO1, EPR-hiCO2, and EPR-loCO) associated with the FeMoco cluster identified during CO turnover, expressed relative to the resting MoFe concentration, are summarized in Tables S2–S4, together with the estimated uncertainties.

#### Control experiments (N_2_ atmosphere)

To establish the distribution of E_*n*_ states accessible under all the e^−^ fluxes, experiments were conducted under N_2_, in the absence of CO (Fig. S1 and Table S1). At 50 : 1 e^−^ flux, only the earliest intermediate E_1_ is observed as established in literature,^98–100^ without any detectable accumulation of E_2_. In contrast, under higher e^−^ fluxes (5 : 1 and 1 : 5), distinct signals corresponding to later E_*n*_ states emerge, including multiple E_2_ isomers (comprising ∼10–20% of the EPR-active population) and low levels of E_4_ (<6%).^101–104^ Across all e^−^ flux regimes, under N_2_ and in the absence of CO, EPR spin quantitation reveals a dominant EPR-silent population (50–80%). These findings underscore the need for complementary spectroscopic techniques, such as IR spectroscopy, to characterize the silent fraction and delineate which E_*n*_ states are competent to interact with CO. The synthesized results from EPR and IR will be presented in a subsequent section.

#### Observations under low and high partial pressures of CO

In the presence of CO, all samples initially display a substantial EPR-silent fraction (∼40–50%) at the earliest time points, which gradually decreases over time to as low as 0–6%, depending on the experimental conditions (Tables S2, S3 and Fig. S2–S6), consistent with previous observations from Orme-Johnson.^39^ The major EPR active species (other than E_0_) at longer time points is characterized as EPR-hiCO (48–78%) irrespective of the reaction conditions. The EPR-hiCO signature comprises two components (EPR-hiCO1: *g* = [2.17, 2.06, 2.05], and EPR-hiCO2: *g* = [2.18, 2.08, 2.05], with larger broadening for EPR-hiCO2). The inclusion and relative populations of these two components were optimized by minimizing the root mean square error (RMSE), as detailed in the SI. At later time points, the combined EPR-hiCO1 and EPR-hiCO2 population accounts for 48–74% (Tables S2 and S3) of the total EPR signal. With our current understanding, we conclude that both EPR-hiCO species contain a bridging µ_2_CO ligand with a terminal CO ligand in different coordination mode (Fe_2_*vs.* Fe_6_, *vide infra*). For simplicity, however, the total hiCO population is reported as the sum of the two species in the FT-IR and EPR correlation section. The accumulation of multiple *S* = 1/2 EPR components beneath the EPR-hiCO signal is further supported by recent EPR photolysis studies employing different light-energy sources for photoirradiation.^52^ However, the identification of the photo irradiated EPR spectra were not well characterized. To achieve a clearer interpretation in this report, we employed a comprehensive simulation approach for the *S* = 1/2 hiCO region together with other EPR-active species. Two distinct EPR-hiCO signals were incorporated into the EPR simulations, which provided a satisfactory fit regardless of the reaction conditions. Notably, the presence of two EPR-hiCO species is validated by their different relaxation properties: EPR-hiCO1 dominates above 6 K temperature, while EPR-hiCO2 dominates below 5 K (Fig. S7). Signals attributed to the previously characterized EPR-loCO species are observed only under low partial pressure CO conditions.

#### Photolysis experiments, EPR

EPR measurements were conducted before and after *in situ* photo-irradiation at 10 K, prepared under conditions to maximize the EPR-loCO or -hiCO species (Fig. S8). Upon photo-irradiation, the EPR-loCO enriched sample showed no significant change in its EPR spectra, even after 40 min of continuous illumination. In contrast, EPR-hiCO enriched samples exhibited clear spectral changes after just 10 min of illumination, with progressive conversion to the EPR-loCO spectral features under both low and high partial pressures of CO. This conversion increased with extended irradiation and was fully reversible upon annealing at 77 K in a liquid nitrogen bath. This annealing step is necessary to achieve the activation energy (∼4 kJ mol^−1^) for the recombination of CO with the FeMoco cluster, which is well documented in the literature.^46^ These observations suggest that the EPR-loCO species is photochemically inert under these conditions, while EPR-hiCO contains a photolabile CO.

#### Effect of additional H_2_, EPR

As H_2_ production is widely accepted as a secondary on-path catalytic process accompanying N_2_ reduction by N_2_ase enzymes, the presence of excess H_2_ is expected to shift the equilibrium between related E_*n*_ and E_(*n*+2)_ states toward the more reduced E_(*n*+2)_ state, in accordance to the LT model (Fig. 1). To investigate effect of H_2_ partial pressure on the appearance EPR-active CO-bound species, EPR measurements were performed under 5 : 1-flux conditions with low CO partial pressure in a 43% H_2_ saturated buffer (Fig. S9, S10 and Table S4). Under these conditions, the *S* = 1/2 EPR-loCO signal was absent at early times points, and an increase in the population of EPR-hiCO was observed compared to equivalent experiments performed in the absence of an H_2_ partial pressure (Fig. S11). These experimental observations suggest that CO binds at multiple E_*n*_ states, the relative populations of which may be shifted by the presence of H_2_, which will stabilize an E_(*n*+2)_ state relative to the corresponding E_*n*_ state.^22^

### FT-IR spectroscopy

FT-IR spectra were collected using two different sample cell systems, as described in Materials and methods: a SF system for earlier timepoints (2–300 s), and a static system for longer timepoints (335–6040 s, Fig. S12). After baseline subtraction (Fig. 4A and S13A for static and Fig. S13B and S14 for SF dataset), each dataset was separately analyzed using NMF to determine the spectral components common to all experimental conditions. Notably, the room-temperature CO turnover FT-IR spectra reveal virtually no discernible features below 1900 cm^−1^ (Fig. S13), with the exception of a weak *ν*(CO) band at approximately 1880 cm^−1^ detected under the high-*p*CO with 1 : 5 e^−^ flux condition, in agreement with a prior stopped-flow study. A further low-frequency band at approximately 1715 cm^−1^, reported by George and coworkers,^51^ was not observed due to limitations of the present experimental setup. The ∼1880 cm^−1^ band has previously been associated with the 1936 cm^−1^ feature based on photolysis studies by Cramer and coworkers.^52^ To enable generalized conclusions to be drawn across all reaction conditions, the NMF analysis presented in this manuscript was restricted to the 1894–1984 cm^−1^ frequency region, considering four distinct *ν*(CO) bands at 1905, 1908, 1936, 1962 cm^−1^, while excluding features below 1894 cm^−1^. The NMF dimensional reduction procedure is somewhat analogous to the well-known principal component analysis (PCA): a small number of spectra (components) are obtained that, when multiplied by a set of weights for each time point (trajectories) and summed, approximate the experimental data (Fig. 4B). Unlike PCA, NMF provides components and trajectories with strictly non-negative values, reflecting the experimental constraint of strictly positive FT-IR intensities and real concentrations. The number of components used in the analysis is chosen manually. We note that NMF additionally constrained to closure and unimodality/monotonicity of weights, often called multivariate curve resolution (MCR), is not appropriate for the present datasets: closure of weights (all weights sum to unity) is inapplicable because multiple species (*e.g.* E_0_ and E_1_) are not detectable, and unimodality/monotonicity (weight trajectories have a single peak or vary monotonically) is inapplicable because multimodal behavior is clearly observed in lo-*p*CO conditions (*vide infra*).

**Fig. 4:**
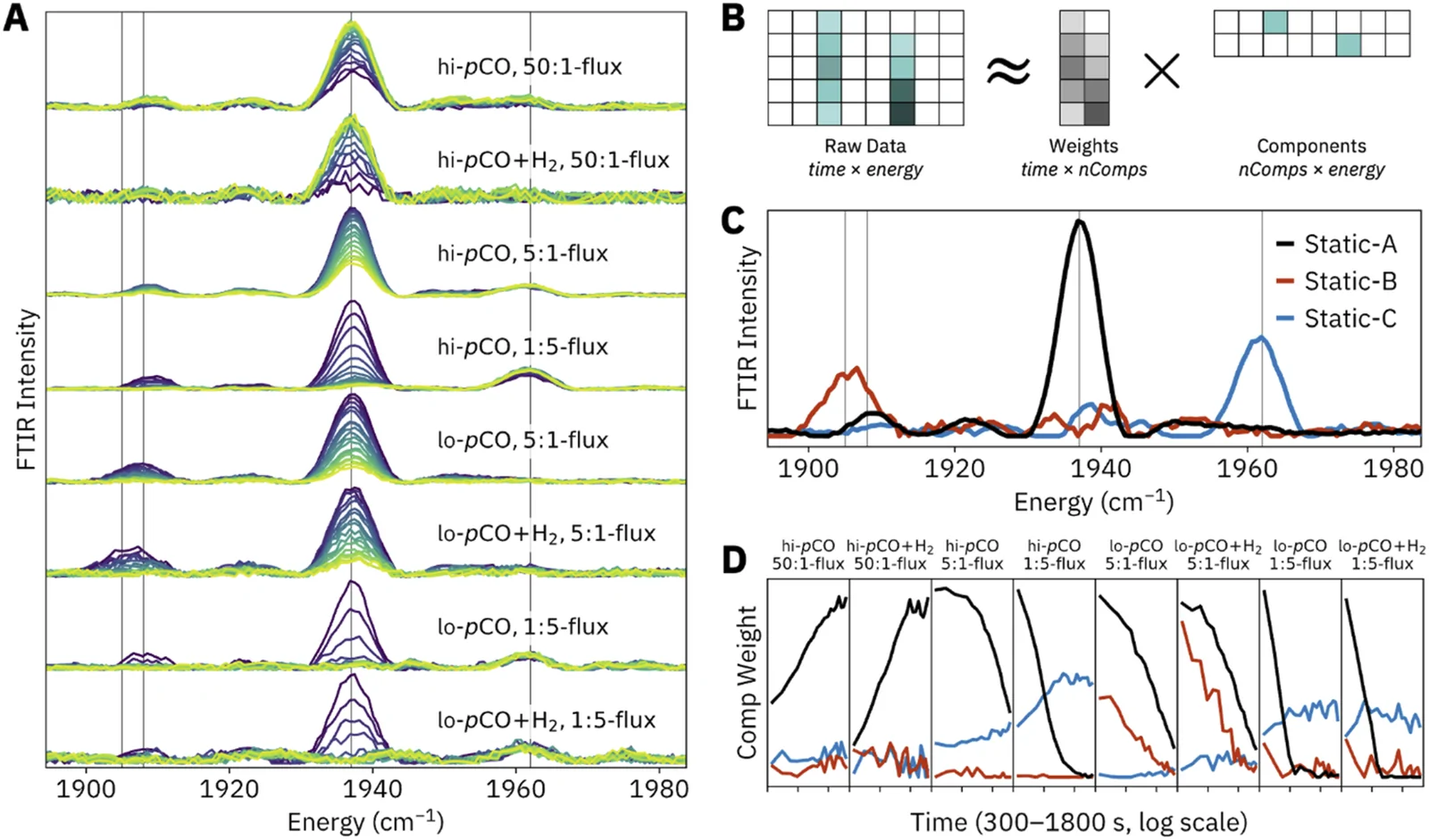
Static FT-IR data and analysis, including (A) baseline-subtracted experimental spectra from 335 s (purple) to 1800 s (green); (B) schematic description of NMF decomposition; (C) three NMF components, termed Static-A, Static-B, and Static-C; and (D) NMF trajectories for the three components plotted in panel C. In panels A and C, thin vertical lines are drawn at 1905, 1908, 1937, and 1962 cm^−1^ to guide the eye. In panel B, the matrices are shown as two-dimensional with the intensity dimension omitted for simplicity.

Here, we present NMF analyses using four components for the static dataset and three components for the SF dataset, numbers chosen based on inspection of the reconstruction error curves and chemical interpretability of the resulting components and trajectories (Fig. S15B and S16B). For each dataset, one component was more noise-like than peak-like, reflecting the time-dependence of the broad background in the experiment and unavoidable Fourier filtering introduced by background subtraction; these components are omitted from the figures in the main text (Fig. 4C and 5A) for clarity but are provided in Fig. S15A and S16A. The analogous NMF components and trajectories for the static and SF dataset are presented in Fig. 4D and 5B. Analogous ^12^CO/^13^CO isotopic labelling experiments to verify the CO assignments are shown in Fig. S17. Based on the NMF analysis of both the stop-flow (SF) and static FT-IR data across the full range of experimental conditions, several general observations can be made regarding the observed spectral features, as summarized below.

**Fig. 5:**
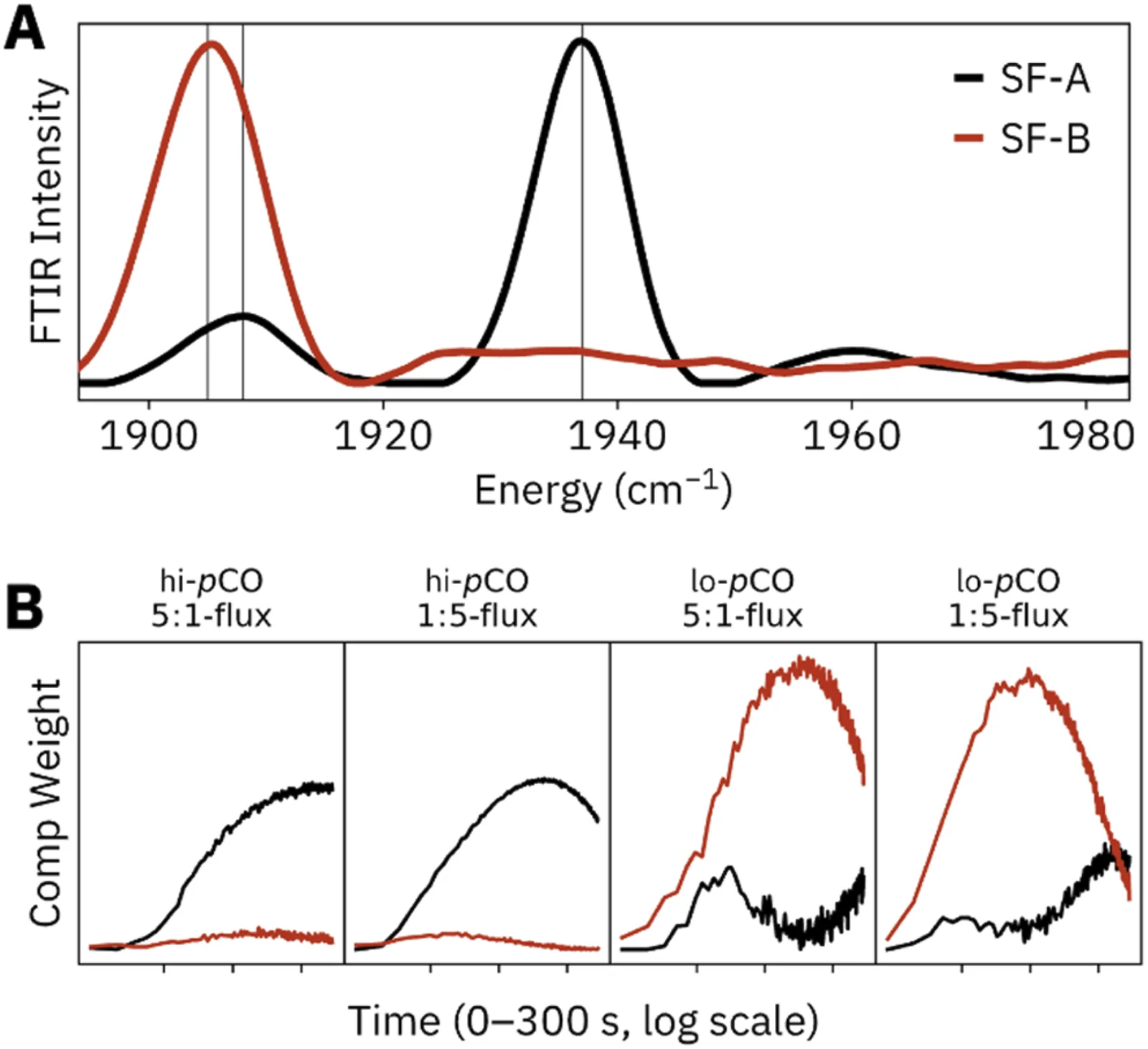
SF FT-IR NMF analysis, including (A) two NMF components, termed SF-A and SF-B; and (B) the trajectories of the components plotted in panel A. In panel A, thin vertical lines are drawn at 1905, 1908, and 1937 cm^−1^ to guide the eye.

#### NMF components of FT-IR spectra

Separate NMF analyses of the static and SF FT-IR spectra reveal two distinct components that are remarkably similar in both datasets: Static-A and SF-A are characterized by two bands at 1908 cm^−1^ and 1937 cm^−1^, while static-B and SF-B are defined by a distinct band at 1905 cm^−1^ (Fig. 4C and 5A). These pairs of similar static and SF NMF components are thus collectively referred to as “Species A” (1908 cm^−1^ and 1937 cm^−1^) and “Species B” (1905 cm^−1^), respectively. The 1937 cm^−1^ feature of Species A typically exhibits the highest peak intensity under most conditions and is observed under all electron flux conditions, though its temporal profile varies. At 50 : 1 e^−^ flux, it appears predominantly at later time points, whereas under higher e^−^ flux, it emerges earlier, suggesting that Species A originates from an earlier E_*n*_ state of the LT cycle. Species B appears predominantly under 5 : 1- and 1 : 5 e^−^ flux, indicating it may correspond to a higher E_*n*_ state that is not populated at low e^−^ flux. Under hi-*p*CO, Species B is observed only at early time points in the SF data, whereas at lo-*p*CO, its contribution becomes more pronounced. Moreover, at lo-*p*CO, for both 5 : 1 and 1 : 5 e^−^ flux, Species A displays a bimodal temporal profile, with Species B peaking between the two maxima of Species A (Fig. 5B). It is notable that separate NMF components and trajectories are associated with intensity centered at 1905 cm^−1^ (Species B) and 1908 cm^−1^ (Species A): although a single feature in this energy region might be identified by visual inspection of any individual FT-IR spectrum, analysis of each complete FT-IR dataset reveals distinct kinetic behavior at slightly lower and higher energies, with intensity at higher energy firmly correlated with intensity at 1937 cm^−1^ in Species A (Fig. 4A and S14).

Species C, defined by a single dominant peak at 1962 cm^−1^, is not observed in the SF data (Fig. 5), but emerges only at longer timescales in the static FT-IR measurements (Fig. 4). At these later stages, Species A and B diminish, and the 1962 cm^−1^ band becomes the dominant feature, the intensity being highly sensitive to experimental conditions. It is most prominent with hi-*p*CO combined with 5 : 1- or 1 : 5-flux. Under lo-*p*CO or 50 : 1-flux conditions, the 1962 cm^−1^ band is not clearly resolved above the background (Fig. 4A).

#### Photolysis experiments, FT-IR

We performed *in situ* photolysis FT-IR experiments at 10 K on frozen samples collected at various time points under different reaction conditions (Fig. S18), and the difference spectra before and after photolysis were utilized to reveal the bridging CO contributions. Representative photolysis spectra for four selected samples are presented in Fig. S18: (i) 1 : 5-flux, 100 s, hi-*p*CO; (ii) 1 : 5-flux, 600 s, hi-*p*CO; (iii) 1 : 5-flux, 3600 s, lo-*p*CO; and (iv) 50 : 1-flux, 3600 s, hi-*p*CO. Upon photo-irradiation, all samples exhibited a sharp decrease in the 1972 cm^−1^ band (corresponding to 1962 cm^−1^ at room temperature), along with a decrease in the ∼1680 cm^−1^ region, and accompanied by the emergence of a new peak at ∼1715 cm^−1^, consistent with previous observations by Cramer and co-workers.^52^ Both the 1680 and 1715 cm^−1^ features are obscured in the SF and static FT-IR measurements because of their overlap with the protein amide bands. The implications of these observations will be discussed further in the EPR data in the following section.

#### Effect of additional H_2_, FT-IR

To gain further insight into the possible distribution and interconversion of E_*n*_ states involved in CO binding, experiments were conducted in the presence of H_2_. At 5 : 1-flux under lo-*p*CO, application of H_2_ partial pressure led to a notable increase in the relative intensity of Species B compared to Species A (Fig. 4D). This shift in spectral distribution suggests a potential redox relationship between the two components, likely corresponding to different E_*n*_ states, related by a two-proton/two-electron reduction to release H_2_. However, under 1 : 5 conditions, no significant accumulation of Species B was observed within our static experimental timeframe (data acquisition started after 300 s), even in the presence of H_2_, likely due to faster reaction rates at this 25-fold higher electron flux condition.

#### Correlation of FT-IR and EPR data

The parallel conditions employed in our FT-IR and EPR studies allow for a direct comparison of results obtained from these complementary techniques. By definition, all FT-IR detected signals correspond to CO-bound species. However, the EPR-silent fraction encompasses odd E_*n*_ states in both CO-bound and unbound forms; therefore, the EPR-silent fraction cannot be quantitatively assigned to E_*n*_-CO, and only a qualitative correlation with the corresponding IR features can be inferred. In contrast, ENDOR studies^42,43,47^ established two EPR-active CO-bound states, EPR-loCO and EPR-hiCO, characterized by distinct *S* = 1/2 signals whose populations can be directly quantified from their spin expectation values obtained from EPR analysis. Thus, if a specific IR component correlates directly with either EPR-hiCO or -loCO states, its intensity is expected to be covariant with the corresponding EPR signal population. Conversely, the absence of such a correlation argues against a direct assignment of that IR component to EPR-hiCO or -loCO.

The results for hi-*p*CO and 50 : 1-flux are presented in Fig. 6. Here, it is important to note that the 1937 cm^−1^ IR feature (major contributor to Species A) decayed at later time points, whereas the EPR active EPR-hiCO species continues to accumulate. This indicates that Species A cannot be the same as EPR-hiCO (an E_2_-CO); rather we identified Species A as E_1_-CO, the only other possible CO binding E_*n*_ state in the 50 : 1-flux condition. We note that time points up to 6040 s were necessary to capture the complete time profile of the 1937 cm^−1^ feature at 50 : 1 e^−^ flux conditions. Comparatively, we considered the time range from 0 to 1800 s under all reaction conditions for NMF analysis to minimize unavoidable background contributions, which increase with the progression of the reactions in the higher flux conditions. Therefore, we plotted the integrated FT-IR intensity centered at 1937 cm^−1^. However, the early intensity profile growth of Species A and the 1937 cm^−1^ band are identical. Both the 1937 cm^−1^ IR feature and the EPR-silent fraction vanish at longer times. This strongly suggests that Species A (comprising 1937 cm^−1^ and 1908 cm^−1^ features) arises from an EPR-silent state.

**Fig. 6:**
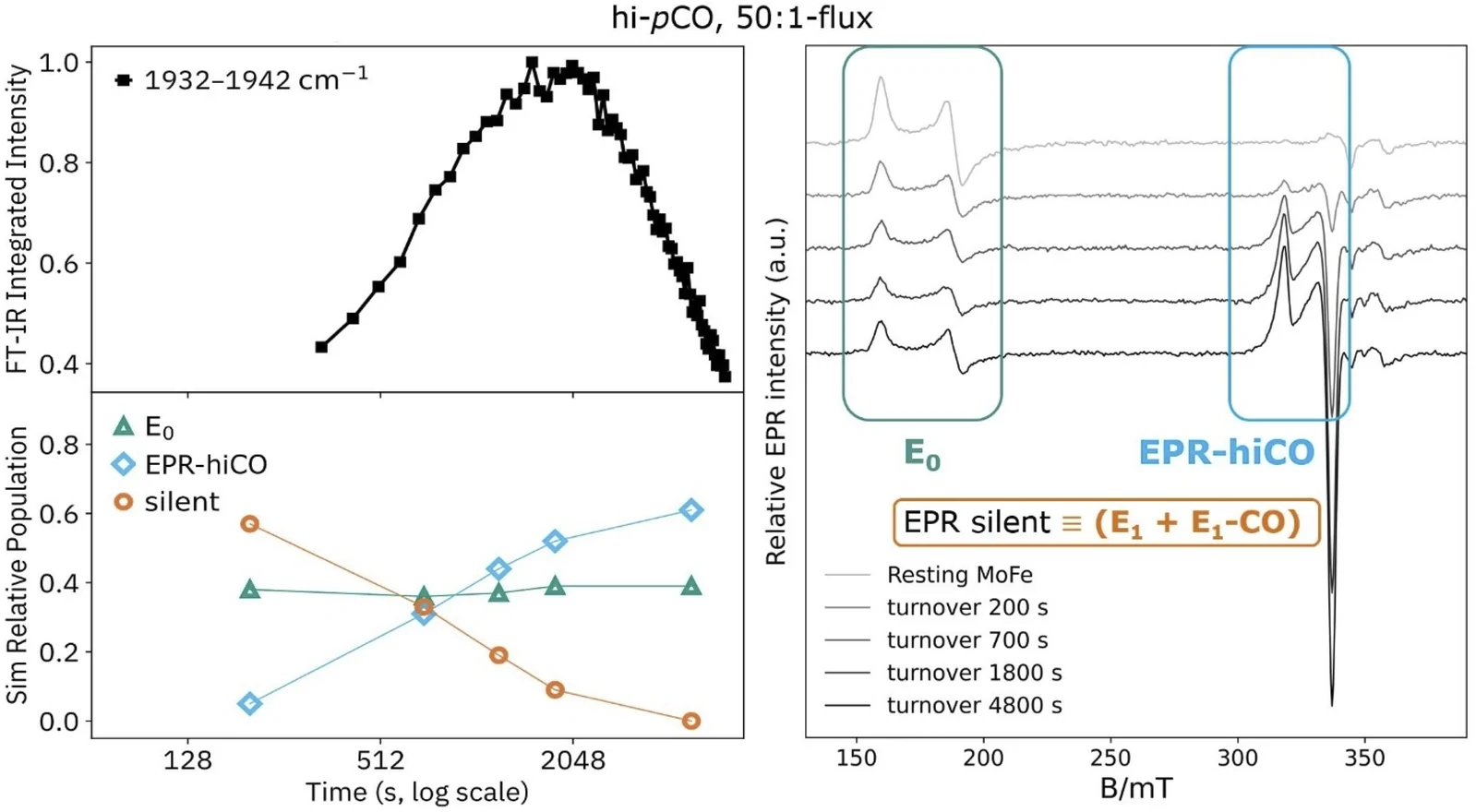
Reaction with hi-*p*CO and 50 : 1-flux, with integrated FT-IR intensity centered at 1937 cm^−1^ (left: top) and relative populations of EPR simulation components (left: bottom) plotted on the same logarithmic time scale. Overlaid EPR spectra recorded at multiple time points under 50 : 1 flux and high-*p*CO conditions (right). The olive, sky-blue, and amber boxes denote the EPR-active E_0_, EPR-hiCO, and EPR-silent species, respectively.

Fig. 7 presents hi-*p*CO data for 5 : 1 and 1 : 5 e^−^ flux conditions. As in the 50 : 1 e^−^ flux case (Fig. 6), Species A (bands at 1908 cm^−1^ and 1937 cm^−1^) decayed at later time points while the EPR-hiCO signal continued to increase until reaching saturation. This further supports the above assignment of the 1908 cm^−1^ and 1937 cm^−1^ features to an E_1_-CO species and that even under higher-flux conditions, which allow access to higher E_*n*_ redox states, CO binding is largely associated with the E_1_ state. Under higher e^−^ flux conditions, Species B (with an IR feature at 1905 cm^−1^) becomes apparent. Species B largely decays with the EPR silent fraction of the sample, indicating that it might correspond to an EPR-silent higher E_*n*_ state. The lo-*p*CO data (*vide infra*) provide further insight into the possible origin of Species B. A key distinction in these e^−^ flux regimes are the emergence of Species C, characterized by the 1962 cm^−1^ band, which appears at later time points. This feature persists together with the presence of EPR-hiCO (under both hi-*p*CO 5 : 1 and 1 : 5 e^−^ flux conditions), indicating that the 1962 cm^−1^ correlates with the EPR-hiCO signals under these conditions. We note that while no clear 1962 cm^−1^ feature was observed in the 50 : 1-flux hi-*p*CO condition as discussed above, photolysis experiments (Fig. S18) under these conditions provide clear evidence for the presence of a terminal CO in this spectral region (*vide infra*). Nevertheless, a quantitative correlation between the EPR-hiCO signal and the 1962 cm^−1^ feature is difficult to make across all flux conditions, and we cannot rule out the presence of more than one terminally bound CO species. This aspect is further discussed in the QM/MM section below.

**Fig. 7:**
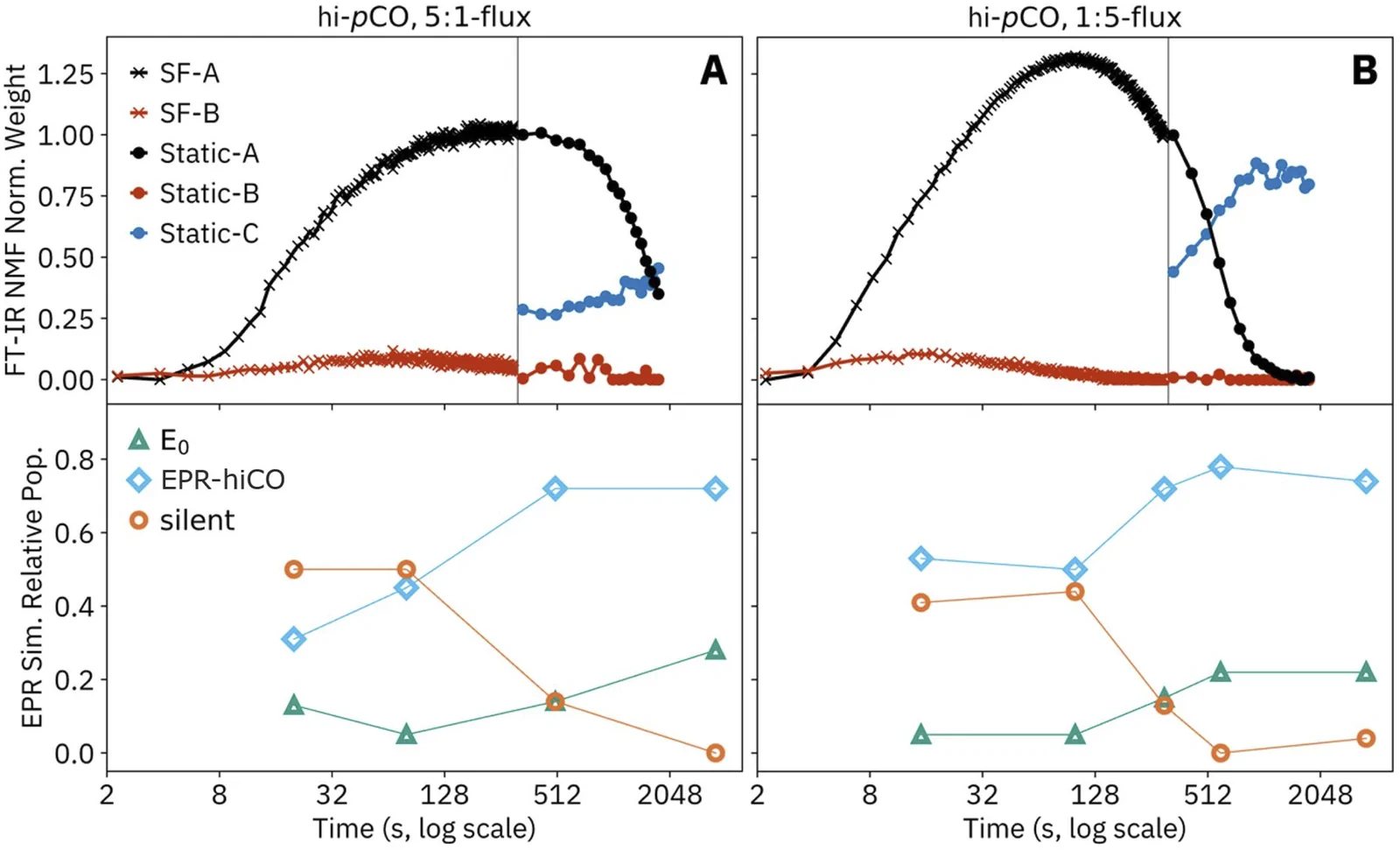
Reactions with hi-*p*CO and 5 : 1-flux (A) or 1 : 5-flux (B), with FT-IR NMF component trajectories (top) and elative populations of EPR simulation components (bottom) plotted on the same logarithmic time scale. The FT-IR NMF components that are associated to a single “species” (*e.g.* SF-A and Static-A, collectively referred to as Species A) are plotted with matching colors. In the top panels, a vertical line divides the SF and static FT-IR datasets.

Fig. 8 presents the lo-*p*CO data for 5 : 1- and 1 : 5-flux. As observed previously, the EPR-hiCO signal reaches a maximum at the longest time points, while both Species A and B have fully decayed in the FT-IR data. Species C persists under 1 : 5-flux conditions (Fig. 8B) but appears to be absent under 5 : 1-flux conditions. Interestingly, both conditions exhibit substantial EPR-hiCO signals, suggesting that the 1962 cm^−1^ feature may be correlated to only a fraction of possible hi-*p*CO states. In this regard, we also note the previously reported difficulty of detecting metallocofactor-bound CO by FT-IR.^105^ Such challenges may further obscure quantitative correlations between the EPR and FT-IR data. For instance, one could envision binding of a terminal CO binding to either Fe2 or Fe6. This possibility is addressed further in the computational section.

**Fig. 8:**
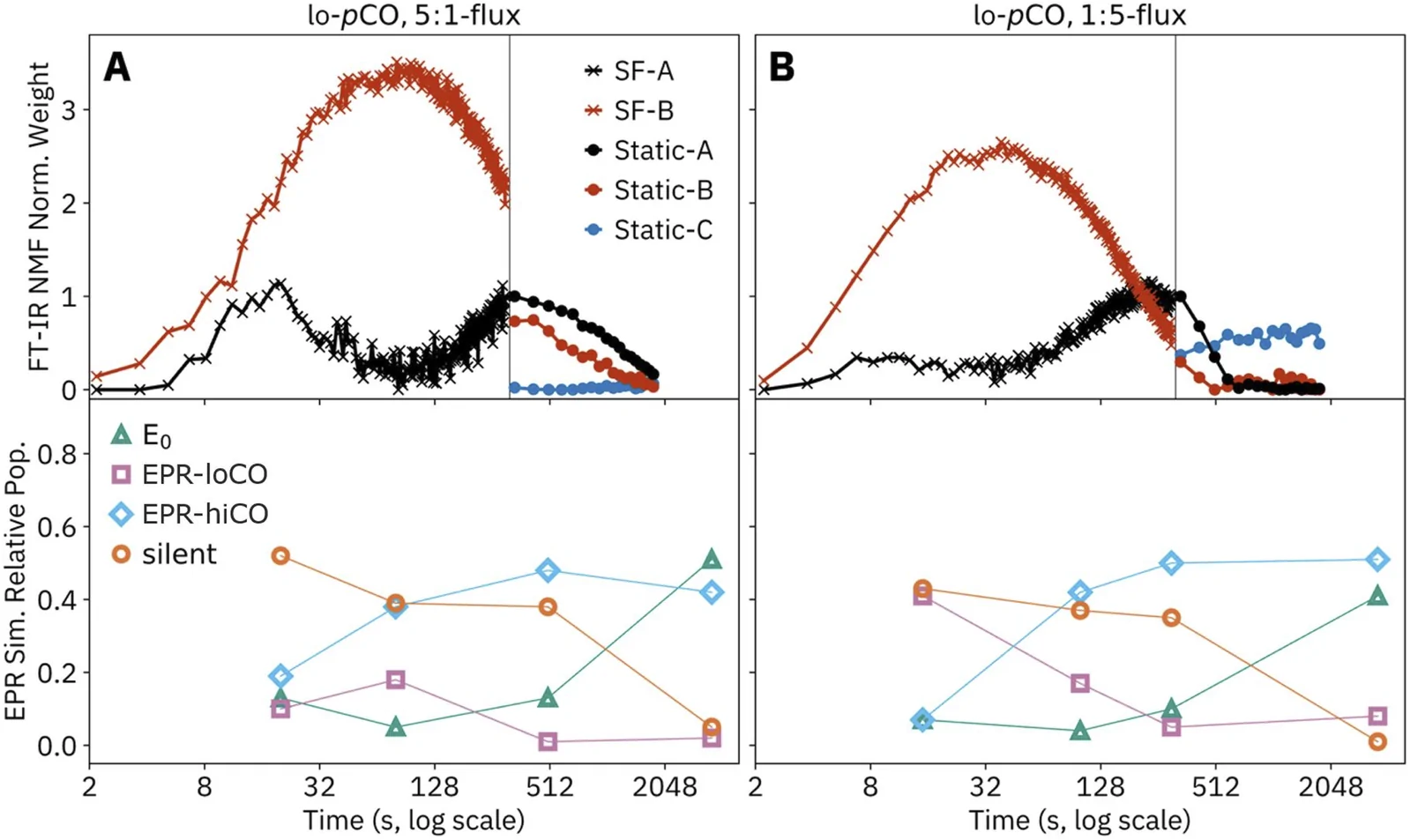
Reactions with lo-*p*CO and 5 : 1-flux (A) or 1 : 5-flux (B), with FT-IR NMF component trajectories (top) and relative populations of EPR simulation components (bottom) plotted on the same logarithmic time scale. In the top panels, the vertical line divides the SF and static FT-IR datasets.

In contrast to the data at hi-*p*CO, at lo-*p*CO the EPR-loCO signal can be observed (Fig. 8). There is no clear correlation of the EPR-loCO population with any of the FT-IR features. However, as lo-CO is generally expected to be a bridging CO species,^43,44,51^ a 1715 cm^−1^ feature is likely to be obscured by the protein amide bands. As noted above, this assignment was confirmed through photolysis measurements. As EPR-loCO and -hiCO signals can be interconverted through photolysis, this indicates these EPR signals likely correspond to the same charge state of the cofactor (Fig. S8).

An important observation under the conditions presented in Fig. 8 is the bimodal temporal behavior of Species A, with Species B peaking at the minima between the two Species A maxima. Given the above-mentioned assignment of Species A to the CO-bound E_1_ state, this behavior could be interpreted as follows: CO initially binds to the E_1_ state. However, at sufficiently high electron fluxes, Mo-nitrogenase is expected to populate more reduced states, most likely E_2_ (EPR-active) and E_3_ (EPR-inactive). Since no E_4_ EPR signal was observed at the earliest time point under any of the reaction conditions in CO turnover (Fig. S2–S6), the involvement of E_4_ at this stage can be excluded. Moreover, as shown in Fig. 8, Species A (E_1_-CO) and Species B appear to be interconnected, with both reaching their maximum populations when the EPR-silent species is maximized in the reaction mixture. Since E_2_ is EPR-active, we consider CO binding to the E_2_ state under the early-turnover conditions unlikely (*vide infra*). Therefore, Species B, which is observed only under higher electron flux conditions, is tentatively assigned as a plausible E_3_-CO state. The experimental data reveal only a single CO stretching frequency (1904 cm^−1^) associated with this species, suggesting that only one CO ligand is bound in this state. This assignment also provides a rationale for the delayed maximum of Species B relative to Species A. Mechanistically, the E_3_-CO state could undergo H_2_ evolution and subsequently return to an E_1_-CO state, thereby accounting for the second increase in the population of Species A. This bimodal behavior is not seen at high CO partial pressure (but notably, Species B is formed and peaks at an earlier time point under 1 : 5 high e^−^ flux compared to moderate 5 : 1 e^−^ flux as expected for a higher reduced CO bound state most likely, E_3_-CO species), presumably because CO binding to E_1_ is strongly favored. Thus, when CO is abundant, E_1_ sites are rapidly saturated with CO, minimizing the presence of free E_1_ and enabling direct reduction of E_1_-CO to form the EPR-loCO and -hiCO species, both of which have a bridging CO. These species are thought to form after the loss of S2B through double protonation and H_2_S release, creating an inhibited state that cannot be reverted by H_2_ evolution. Under higher electron-flux conditions, we observe the formation of a second CO-bound species that is kinetically and spectroscopically distinct from the E_1_-CO state and is best gauged to CO binding at the E_3_ level. This possibility is supported by the flux-dependent emergence of this species and, importantly, by its sensitivity to partial H_2_ pressure, which selectively modulates the relative rates of formation and decay of the E_1_- and E_3_-like bound CO species. The ability of H_2_ to differentially perturb these populations is consistent with a redox relationship between the two states and provides strong evidence that both E_1_ and E_3_ are competent for CO binding under turnover conditions (Fig. 4D).

### QM/MM calculations

To better understand our spectroscopic results, we calculate the accessible CO-bound isomers of FeMoco in its E_1_ and E_2_ redox states, considering both terminal and bridging binding conformations at the Fe2 and Fe6 sites. We further consider structures in the E_2_ state with both terminal and bridging CO. The QM/MM optimized geometries, highlighting the primary coordination sphere of the Fe2 and Fe6 centers are shown in Fig. 9, and the calculated CO binding energies (Δ*E*_CO_) and vibrational frequencies in Table 3. The isomers' relative stabilities (Δ*E*_isomer_) are reflected in their Δ*E*_CO_ energies because they're referenced to the same E_*n*_ state, as detailed in the computational procedure above. Each structure is referenced to the lowest-energy broken symmetry wavefunction, with the associated spin populations at the metal centers summarized in Tables S5 and S6. The BS-solutions exhibit distinct distributions of oxidation states, resulting in unequal CO-binding energies that favor coordination to the more reduced Fe-centers. We have previously detailed the CO-bound isomers of the E_1_ state of FeMoco, whose CO ligands can attractively coordinate terminal to the Fe2 and Fe6 position.^59^ Our calculations aren't extended to the E_3_ state because of the large number of possible hydride-containing and protonated isomers which could bind CO and very limited experimental data available for this EPR silent intermediate.^99,100^ We note that Species B's 1905 cm^−1^ CO vibrational mode, whose H_2_ pressure dependence could suggest an E_3_ state, would be consistant with a terminal CO binding mode at a reduced Fe-center, similar to the [E_1_](CO)_Fe6_ structure.

**Fig. 9:**
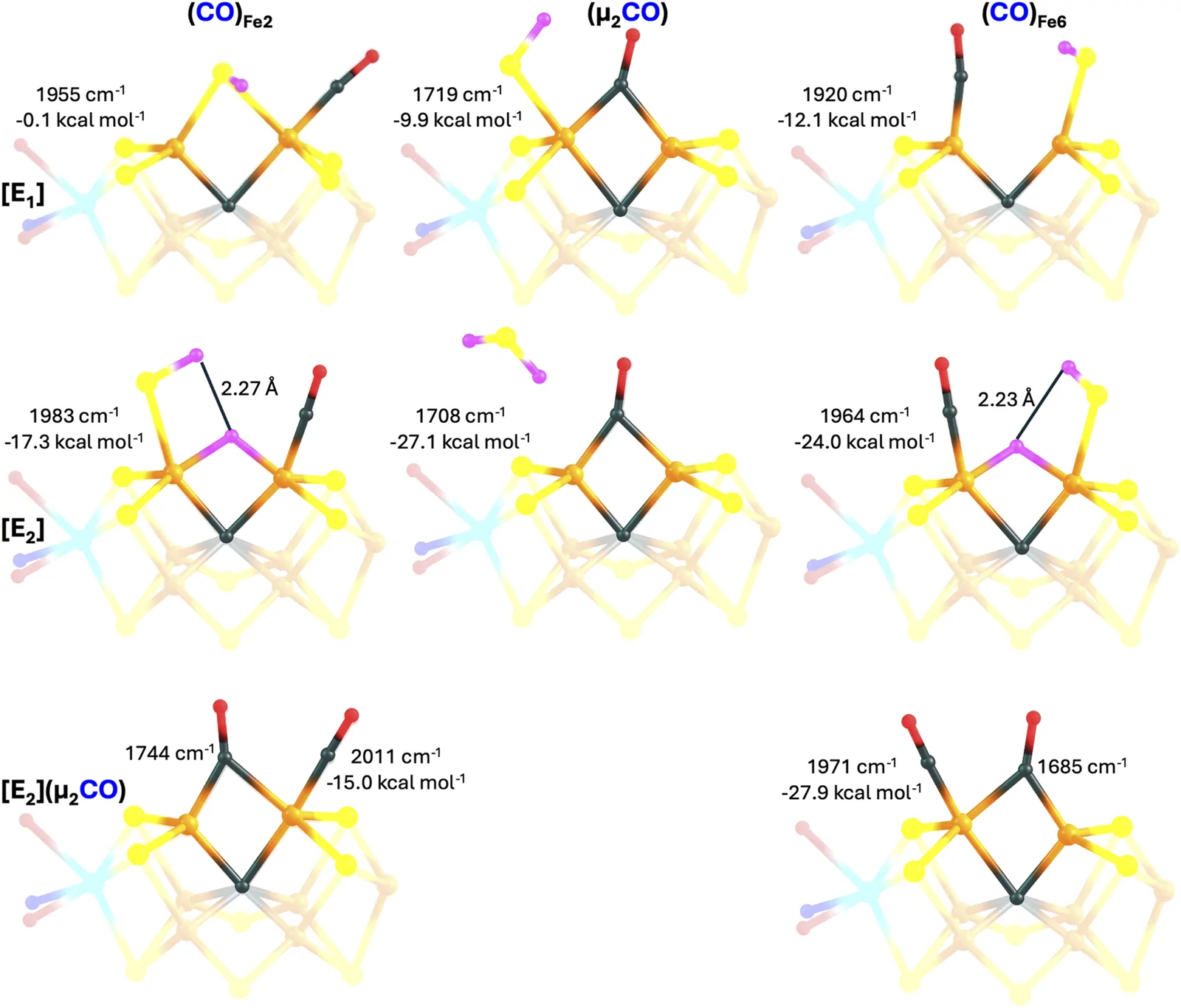
The QM/MM optimized E_1_ (top) and E_2_ (middle) CO-bound isomers that coordinate the Fe2 (left), µ_2_-bridging (center), and Fe6 (right) sites. The structures for a second CO-binding to the terminal Fe2 and Fe6 sites is shown with respect to the [E_2_](µ_2_CO) structure (bottom). The calculated *ν*(CO) mode and electronic binding energy are provided. The primary coordination sphere of the Fe2 and Fe6 sites are highlighted for clarity. When applicable, the distance between a hydride and proton is provided.

**Table 3:** The calculated electronic CO binding energies (Δ*E*_CO_), relative isomer energies (Δ*E*_isomer_) and C–O vibrational frequencies (*ν*_CO_) of the QM/MM optimized CO bound isomers of FeMoco. The Fe-site that binds CO is specified in the state's notation

State	BS-solution	Δ*E*_CO_ (kcal mol^−1^)	Δ*E*_isomer_ (kcal mol^−1^)	*ν*_CO_ calculated (cm^−1^)
[E_1_](CO)_Fe2_	BS-235 (*M*_s_ = 2)	−0.1	12.0	1955
[E_1_](µ_2_CO)	BS-346 (*M*_s_ = 1)	−9.9	2.2	1719
[E_1_](CO)_Fe6_	BS-346 (*M*_s_ = 2)	−12.1	0.0	1920
[E_2_](CO)_Fe2_	BS-147 (*M*_s_ = 1/2)	−17.3	9.8	1983
[E_2_](µ_2_CO)	BS-235 (*M*_s_ = 1/2)	−27.1	0.0	1708
	BS-147 (*M*_s_ = 1/2)	−26.4	0.7	1778
[E_2_](CO)_Fe6_	BS-235 (*M*_s_ = 1/2)	−24.0	3.1	1964
[E_2_](µ_2_CO)(CO)_Fe2_	BS-247 (*M*_s_ = 1/2)	−15.0	7.8	1744, 2011
[E_2_](µ_2_CO)(CO)_Fe6_	BS-235 (*M*_s_ = 1/2)	−27.9	0.0	1685, 1971

We have shown in previous spectroscopic and computational studies that the µ_2_S2B site is protonated in the E_1_ state of FeMoco,^99,106^ whose lability can adopt a terminal coordination mode to accommodate substrate binding.^64,106^ The Fe2 and Fe6 sites in the most stable E_1_ configuration differ in both their oxidation states, Fe6 being more reduced, and in their local protein environments, with the His195 residue sterically hindering Fe2. As a result, Fe2 exhibits a very weak CO binding energy of −0.1 kcal mol^−1^ and binds CO axially within a trigonal bipyramidal geometry, showing a 1955 cm^−1^ vibrational mode. The cluster preferentially reorganizes by −9.8 kcal mol^−1^, in which the protonated sulfide bridge opens, adopting a terminal binding mode at Fe6, allowing the µ_2_CO binding conformation. We note that the E_2_(CO)_Fe2_ structure freely optimized to the E_2_(µ_2_CO) when the *M*_s_ state is changed from 2 to 1, which suggests a small reorganization barrier. However, because the E_2_(CO)_Fe2_ and E_2_(µ_2_CO) isomers have distinct *M*_s_ states the transition state that connects them can't be directly calculated without accounting for the spin barrier.^107^

This CO bridge forms strong Fe–C σ-bonds and serves as a hydrogen bond acceptor to His195, weaking the C–O stretching frequency from 1955 to 1719 cm^−1^ whose degree of activation is consistent with model Fe-complexes.^55,108^ We do not find stable structures where the terminal SH dissociates from Fe6, suggesting the S2B remains bound to the cofactor's E_1_ state. At the Fe6 center, CO binds in a tetrahedral coordination geometry, with a stretching frequency of 1920 cm^−1^, providing a comparable stabilization of −12.1 kcal mol^−1^. Reported by Suess, CO-bound Fe–S clusters with an [MFe_3_S_4_]^+^ (M = Fe and Mo) charge state have C–O vibrational modes of approximately 1905 cm^−1^.^54,109^

In the more reduced E_2_ state, both terminal CO isomers form a bridging hydride, formally oxidizing the coordinated Fe2 and Fe6 sites and increasing their respective *ν*(CO) frequencies to 1983 and 1964 cm^−1^. As in the E_1_ structures, CO binds more strongly at Fe6 (−24.0 kcal mol^−1^) than at Fe2 (−17.3 kcal mol^−1^). An average distance of 2.25 Å between the µ_2_-hydride and the proton of the terminal SH group indicates a potential pathway for regenerating the cofactor's E_0_ state through H_2_ formation. This has been previously detailed for FeMoco's E_2_ state, reporting an approximate activation energy of 20 kcal mol^−1^.^110,111^ The activation barrier was attributed to the dissociation of the S2B–H bond that shouldn't be directly impacted by the CO binding to a separate Fe-center, as shown in the [E_2_](CO)_Fe2_ and [E_2_](CO)_Fe6_ structures.^112^ We note, that while FeMoco's E_0_ state doesn't directly bind CO, the terminal CO groups could remain coordinated following H_2_ production, giving rise to the [E_0_](CO)_Fe2_ and [E_0_](CO)_Fe6_ structures that we detailed in our previous QM/MM study.^59^ The corresponding [E_0_](µ_2_CO) isomer proposed for the analogous FeVco lo- and hi-CO states^62^ isn't accessible by this mechanism of H_2_ production because S2B remains in the cofactor.

In the [E_2_](µ_2_CO) isomer, the terminal SH group at Fe6 and the resulting H_2_S dissociates during optimization, suggesting a barrierless process. The properties reported for the [E_2_](µ_2_CO) isomer in Fig. 5 and Table 3 include H_2_S in the QM-region. We note that, while the BS-235 and -147 solutions are nearly degenerate, their respective C–O vibrational modes occur at 1708 and 1778 cm^−1^. The BS-235 optimized geometry features an approximately symmetric µ_2_CO bridge, consistent with the loCO crystal structure,^44^ while its more activated C–O vibrational mode is more consistent with our current photolysis data. Comparatively, the BS-147 solution adopts a linear semibridging conformation, whose respective C–Fe2 and -Fe6 bond distances are 1.76 and 2.13 Å.^113^ The H_2_S is 3.5 Å away from the Fe6 center and hydrogen bonds with a carboxylate group of the homocitrate, which minimally impacts the CO vibration +4 cm^−1^ when compared to removing the H_2_S leaving group from the calculations. The analogous structure where H_2_S is formed at Fe2 is energetically unfavorable because of the steric repulsion between His195 and the elongated Fe2–S2B bond. Similarly, structures that form both a µ_2_-hydride and µ_2_-carbonyl are energetically unfavorable. As [E_2_](µ_2_CO) isomer lacks hydrides, it cannot produce H_2_ directly to return to the E_0_ resting state or reduce the coordinated CO. The dissociation of H_2_S from the cofactor in its E_2_ state requires a substrate that can bind to the resulting reduced and coordinatively unsaturated Fe-sites. While our calculations suggest that H_2_S, rather than HS^−^, is the preferred leaving group for S2B, the dissociated S2B is best modelled crystallographically as HS^−^ in the sulfide holding pocket.^62^

The steric constraints associated with binding a second CO at Fe6 promote further separation of H_2_S from the cofactor, stabilizing the system by −27.9 kcal mol^−1^. We note that the Δ*E*_CO_ values for the second CO binding are referenced with respect to the [E_2_](µ_2_CO) structure where the dissociated H_2_S is removed. The [E_2_](µ_2_CO)(CO)_Fe6_ isomer is consistent with the reported hi-CO crystal structure.^45^ The respective µ_2_-and terminal-*ν*(CO) frequencies are 1685 and 1970 cm^−1^, consistent with FT-IR photolysis studies of samples prepared under hiCO conditions^56^ and the NMF component Static C of the present study. The second CO binds weaker to Fe2 (−15.0 kcal mol^−1^) in the [E_2_](µ_2_CO)(CO)_Fe2_ structure, resulting in less activated *ν*(CO) frequencies of 1744 and 2011 cm^−1^ at the respective µ_2_- and terminal-CO ligands. We note that the distinct EPR-hiCO1 and -hiCO2 signals observed could possibly be attributed to these distinct terminal CO binding modes or the low-lying conformations of the µ_2_-binding mode as symmetric or linear, as detailed above.

## Discussion

We summarize our mechanistic assignment for CO binding to FeMoco in Fig. 10 and Table 4. The gradual increase of the EPR-hiCO signatures with a decrease in Species A in 50 : 1-flux conditions confirms that the EPR silent E_1_ state is responsible for initial CO binding. Species A in our NMF analysis of the IR spectra corresponds to the CO bound E_1_ state, whose 1937 and 1908 cm^−1^ vibrational signatures reflect two distinct terminal CO binding sites. Notably, while previous studies have proposed these correspond to one state of the cofactor with two terminally bound CO, we propose these bands correspond to two unique single CO-bound species differentiated by binding at either Fe2 or Fe6. Even under higher-flux conditions, which allow access to higher E_*n*_ redox states, Species A persists, indicating that CO binding is largely associated with the E_1_ state. We calculate that the E_1_ state can spontaneously bind CO, whose [E_1_](CO)_Fe2_ and [E_1_](CO)_Fe6_ isomers we relate to the respective 1937 and 1908 cm^−1^ vibrations of Species A observed in the experiments. Because of the greater stability of the bridging species, [E_1_](CO)_Fe2_ should reorganize to [E_1_](µ_2_CO), which is consistent with the decay of the 1937 cm^−1^ feature that isn't replaced by another terminal *ν*(CO) frequency. Indeed, previous studies by Thorneley and George reported the delayed appearance of a 1715 cm^−1^ band that displays continued accumulation after the decay of the 1937 cm^−1^ feature, consistent with a [E_1_](CO)_Fe2_ to [E_1_](µ_2_CO) interconversion.^51^

**Fig. 10:**
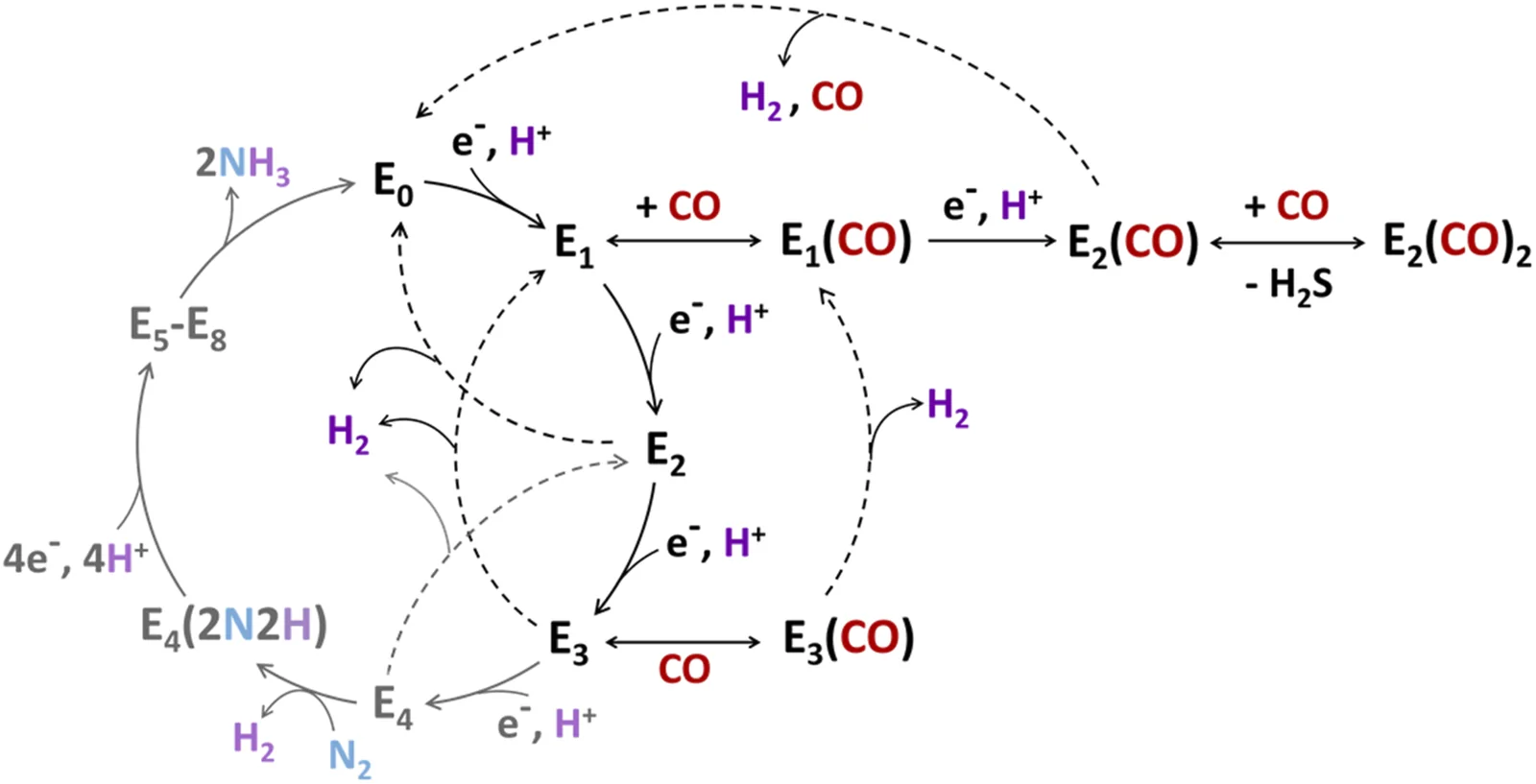
The mechanistic relationship between the Lowe–Thorneley cycle for nitrogen fixation (left) and CO reduction (right) from CO binding in FeMoco. The experimental and calculated properties of the CO bound isomers are provided in Table 4 below. The suffix after all E states indicate the number of proton and electron consumption and the suffix after the parenthesis indicate the number of bound CO molecule.

**Table 4:** The consolidated IR, EPR, and QM/MM data for the states listed in Fig. 10

State	Structure	*ν*_CO_, cm^−1^ experimental (Calculated)	Spin state experimental (calculated)
[E_1_](CO)	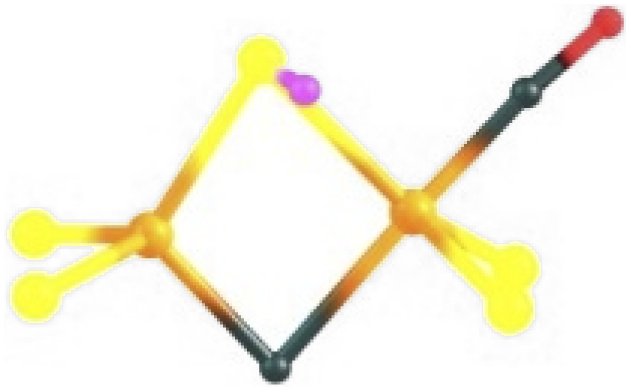	Static-A: ∼1937 SF-A: ∼1937 (1955)	Silent (*M*_s_ = 2)
	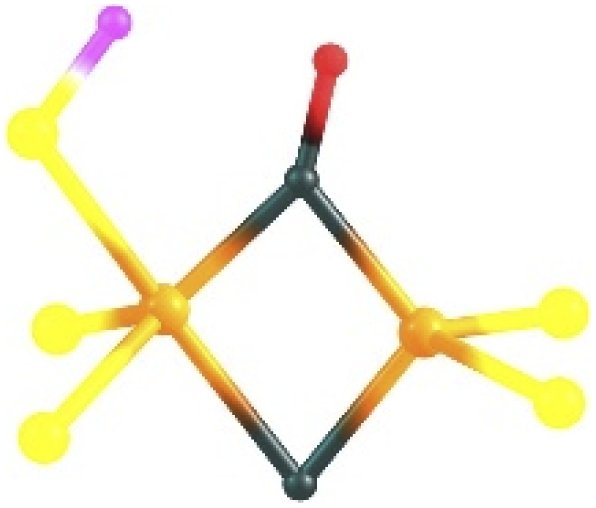	Obscured by amide (1719)	Silent (*M*_s_ = 1)
	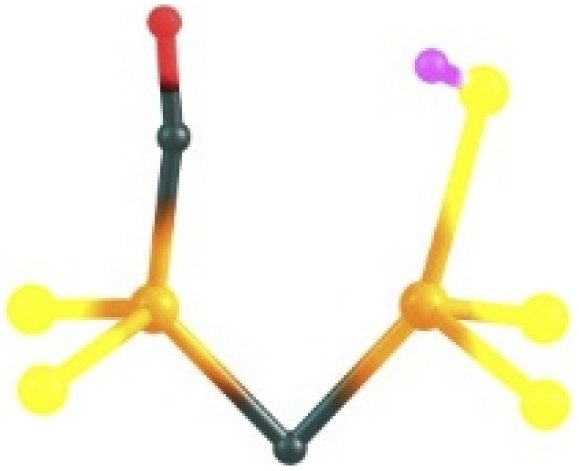	Static-A: ∼1908 SF-A: ∼1908 (1920)	Silent (*M*_s_ = 2)
[E_2_](CO)	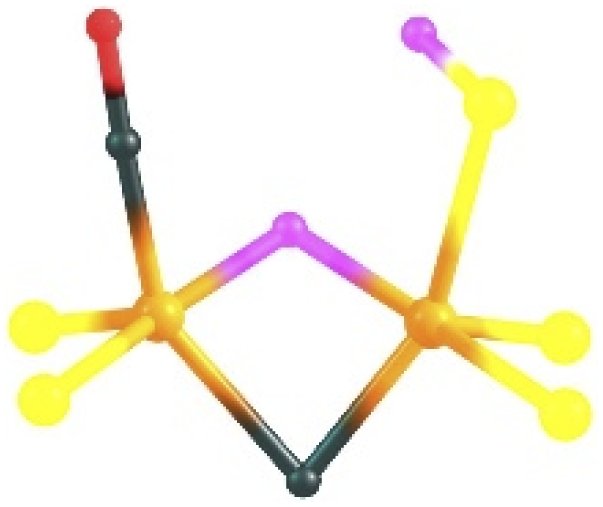	Relaxes to E_0_ state through H_2_ formation (1964)	(*M*_s_ = 1/2)
	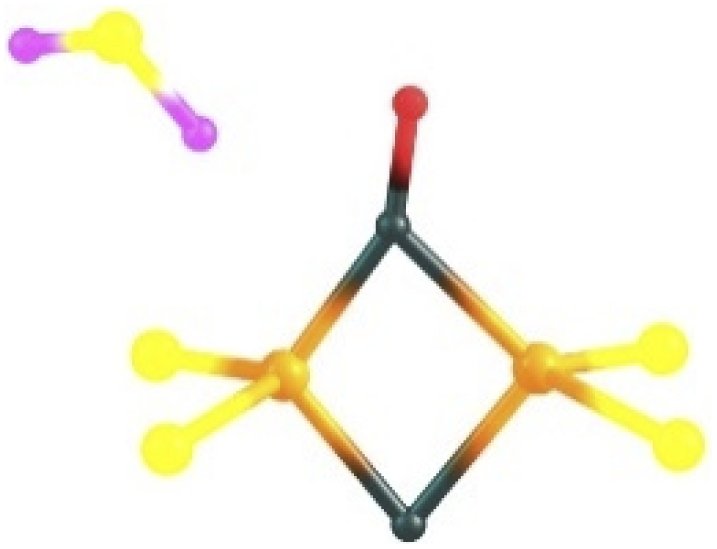	Photolysis: ∼1715 (1708)	EPR-loCO: *S* = 1/2 (*M*_s_ = 1/2)
[E_2_](CO)_2_	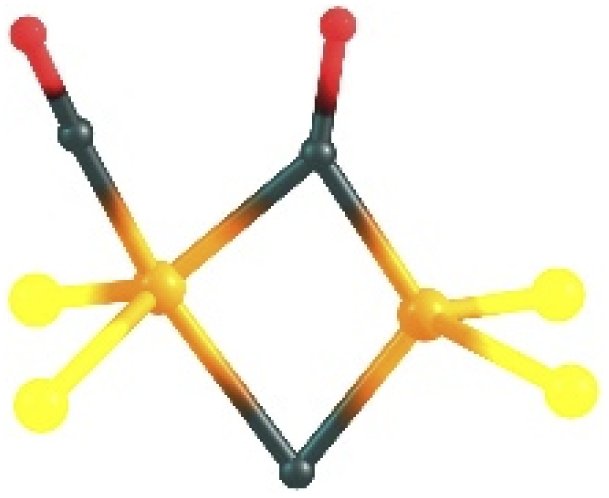	Static-C: 1962 photolysis: ∼1685, 1972 (1685, 1971)	EPR-hiCO: *S* = 1/2 (*M*_s_ = 1/2)
[E_3_](CO)	(−)	Static-B: 1905 SF-B: 1905 (−)	Silent (−)

Notably, despite extensive exploration across a wide range of electron-flux and CO partial-pressure conditions, we haven't found any spectroscopic evidence for direct CO binding to the E_2_ state. Rather, our evidence suggests the formation of EPR-active CO-bound species is preceded by the formation of [E_1_](CO) species. While our QM/MM calculated CO binding energies are more favorable for the E_2_ state, the E_1_ states' CO affinities could be sufficiently high so that CO binding occurs before any appreciable accumulation of E_2_. Alternatively, the E_2_ intermediate may be too short-lived to bind CO, the kinetics for H_2_ production by the E_2_ state recently reported as orders of magnitude faster than the E_3_ state.^114^ Such kinetic bias would strongly disfavor the buildup of a population of directly formed [E_2_](CO) species, even if transient binding were possible. The proximal hydride and proton groups in the calculated structures of [E_2_](CO)_Fe2_ and [E_2_](CO)_Fe6_ suggest that both isomers can return to the E_0_ resting state by H_2_ production, which is consistent with maintained proton reduction of FeMoco in CO environments.

Compared to [E_1_](µ_2_CO), the [E_2_](µ_2_CO) isomer has a doubly protonated S2B whose dissociation is consistent with the crystallographic structures of FeMoco.^44^ The absence of a pathway for H_2_ production is consistent with the stability of FeMoco's CO inhibited species and the marginal impact of H_2_ on the relative fractions of the EPR-active species.

Consistent with Einsle and co-worker's mechanistic proposal, the EPR-hiCO species is a catalytic dead-end that displaces the CO bound cofactor from possible CO reduction and hydrocarbon formation.^62^ However, during turnover, an EPR-silent CO-bound E_3_ like state was identified as Species B through NMF analysis, exhibiting a terminal *ν*(CO) band at 1905 cm^−1^. A distinct equilibrium between Species B and Species A (a different terminal CO-bound E_1_ state) was established by analyzing their relative populations in the presence and absence of H_2_ in the medium. Together, these observations suggest that while CO binding is initiated predominantly at E_1_, additional CO-bound intermediates become accessible during turnover and may participate in the mechanism of CO inhibition of FeMoco.

## Conclusion

Our report demonstrates a comprehensive understanding of CO reactivity towards Mo N_2_ase enzyme, elucidating a detailed mechanism using a combination of freeze quench CW-EPR and SF/FQ FT-IR spectroscopies, further supported by QM/MM calculations, providing essential insights into N_2_ase function. Using a low electron flux (50 : 1-flux), we have established that CO binding is strongly associated with the one-proton, one-electron reduced E_1_ state of FeMoco. By integrating experimental and theoretical findings, two potential CO binding sites, Fe6 and Fe2, has been identified with terminal CO bound vibrational frequencies of 1908 and 1937 cm^−1^, respectively. Further considering three different electron flux regimes, two possible transient EPR silent CO binding E_*n*_ states (E_1_ and E_3_) are characterized. Our orthogonal spectroscopic approach reveals that, during the early turnover state, the major fraction of the EPR-silent species corresponds to the CO-bound, EPR-inactive E_1_ and E_3_ states. However, after prolonged turnover (∼1 hour), the EPR active species reappear with the EPR-hiCO species being dominant and a smaller fraction of the resting E_0_ state, regardless of the reaction conditions. The EPR-hiCO species form subsequent to the EPR-loCO state and are assigned to a µ_2_CO–E_2_ configuration. The EPR-hiCO signal comprises a mixture of two distinct paramagnetic species, designated EPR-hiCO1 and EPR-hiCO2, which differ in their spin relaxation behavior, as supported by simulation. Correlated EPR and FT-IR photolysis experiments indicate that the EPR-hiCO species are associated with a doubly CO-bound E_2_ state, characterized by vibrational bands at 1962 (corresponding to 1972 cm^−1^ at 10 K) and 1680 cm^−1^. However, the 1962 cm^−1^ feature does not quantitatively correlate with the EPR-hiCO signal across all flux conditions, suggesting that an additional terminal CO-bound species (*e.g.*, at Fe2 *versus* Fe6) may contribute. Alternatively, a non-CO ligand cannot be fully excluded, although computational results provide no support for this possibility. Finally, by combining the experimental and theoretical results, we propose that the bridging EPR-loCO state forms first during CO turnover by Mo N_2_ase, followed by formation of the EPR-hiCO species. The latter represents the thermodynamic sink of the reaction and corresponds to the inhibited form of the enzyme. In contrast, a terminal E_2_-CO species (prior to the formation of a bridge) could provide a potential pathway for enzyme regeneration. Based on our current understanding, we hypothesize that an increased propensity for terminal CO binding, rather than bridging CO coordination, together with enhanced stability of this intermediate, may enable further reduction of CO. This scenario could help explain the catalytic activity of V and Fe N_2_ase toward CO reduction. However, further experimental investigations are required to test and validate this hypothesis.

## Author contributions

AR, CVS, and SD conceived and designed the study. Expression and purification of nitrogenases were performed by LD and KS, and sample preparation was carried out by AR, KS, and LD. Room-temperature FTIR and EPR data collection, as well as EPR simulations, were performed by AR. Data analysis was conducted by AR, ZM, KS, CVS, and SD. NMF analysis was performed by ZM. Computational studies were carried out by JPJ. Low-temperature FTIR measurements were performed by CL and IZ. SD supervised the study. The manuscript was written by AR, JPJ, KS, ZM, CVS, and SD and was reviewed and edited by all authors.

## Conflicts of interest

There are no conflicts to declare.

## Supplementary Material

SC-OLF-D6SC05535D-s001

## Data Availability

All experimental and computational data, as well as example ORCA input files, are available at the Edmond open data repository: https://doi.org/10.17617/3.KLBC1T. Supplementary information (SI) is available. See DOI: https://doi.org/10.1039/d6sc05535d.
