# Supplementary material for: Redox-state-dependent CO binding in MoFe nitrogenase: insights from EPR, IR, and QM/MM analysis
Source: Chem Sci. 2026 Oct 7. Online ahead of print. doi: 10.1039/d6sc05535d (PMC13670219; doi:10.1039/d6sc05535d)
Supplement: Supplementary file 1 [file SC-OLF-D6SC05535D-s001.pdf]

Supplementary Information (SI) for Chemical Science.
This journal is © The Royal Society of Chemistry

Supporting Information

for

Redox-State-Dependent CO Binding in MoFe Nitrogenase: Insights from EPR, IR, and QM/MM Analysis

Atanu Rana,[+a] Justin P. Joyce,[+a] Zachary Mathe,[+a] Casey Van Stappen,[a] Kushal Sengupta,[a] Christian Lorent,[b] Ingo Zebger,[b] Laure Decamps,[a] Serena DeBeer*[a]

[a] Max Planck Institute for Chemical Energy Conversion, Stiftstrasse 34-36, 45470 Mülheim an der Ruhr, Germany

[b] Institut für Chemie, PC14, Physikalische und Biophysikalische Chemie, Technische Universität Berlin, Strasse des 17. Juni 135, 10623 Berlin, Germany

email: serena.debeer@cec.mpg.de

[+]These authors contributed equally

Table of Content:

Figure S1 -- S3

Figure S2 -- S4

Figure S3 -- S5

Figure S4 -- S6

Figure S5 -- S7

Figure S6 -- S8

Figure S7 -- S9

Figure S8 -- S10

Figure S9 -- S11

Figure S10 -- S12

Figure S11 -- S13

Figure S12 -- S14

Figure S13 -- S15

Figure S14 -- S16

Figure S15 -- S17

Figure S16 -- S18

Figure S17 -- S19

Figure S18 -- S20

Figure S19 -- S21

Table S1 --- S22

Table S2 --- S23

Table S3 --- S24

Table S4 --- S25

Table S5 --- S25

Table S6 --- S26

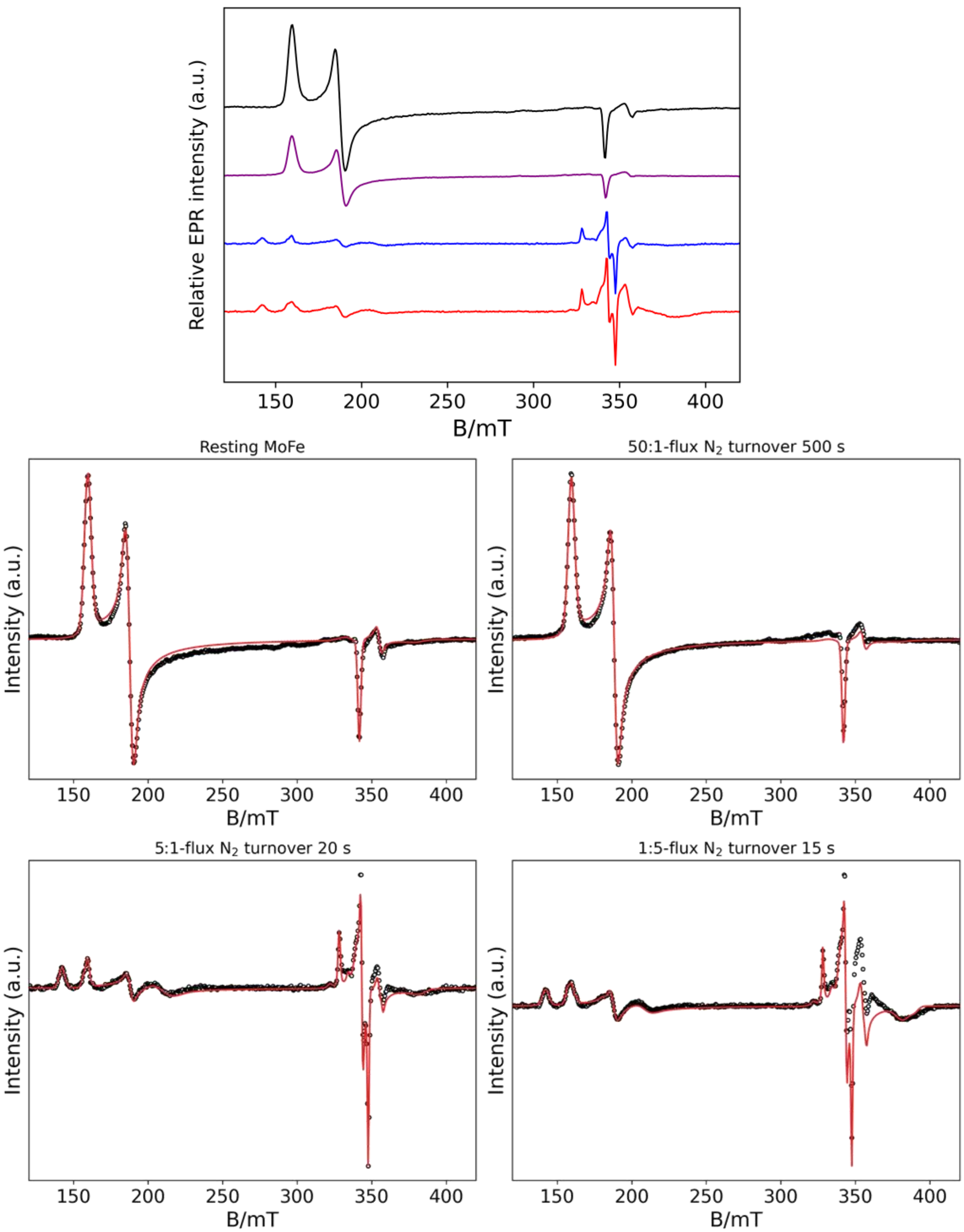

Figure S1: The EPR spectra overlay (top) resting (black) and three different nitrogen(N_2) turnover samples at [MoFe]:[FeP] electron fluxes of 50:1 (purple), 5:1 (blue), and 1:5 (red). Rest the experimental EPR spectrum (depicted as a black circle) alongside its corresponding fits (illustrated as a red line) in different time points as mentioned above the individual plot, in addition to the resting FeMoco. In the higher-flux conditions an additional rhombic $S = 1/2$ EPR appear due to presence of oxidized FeP (FeP^+).

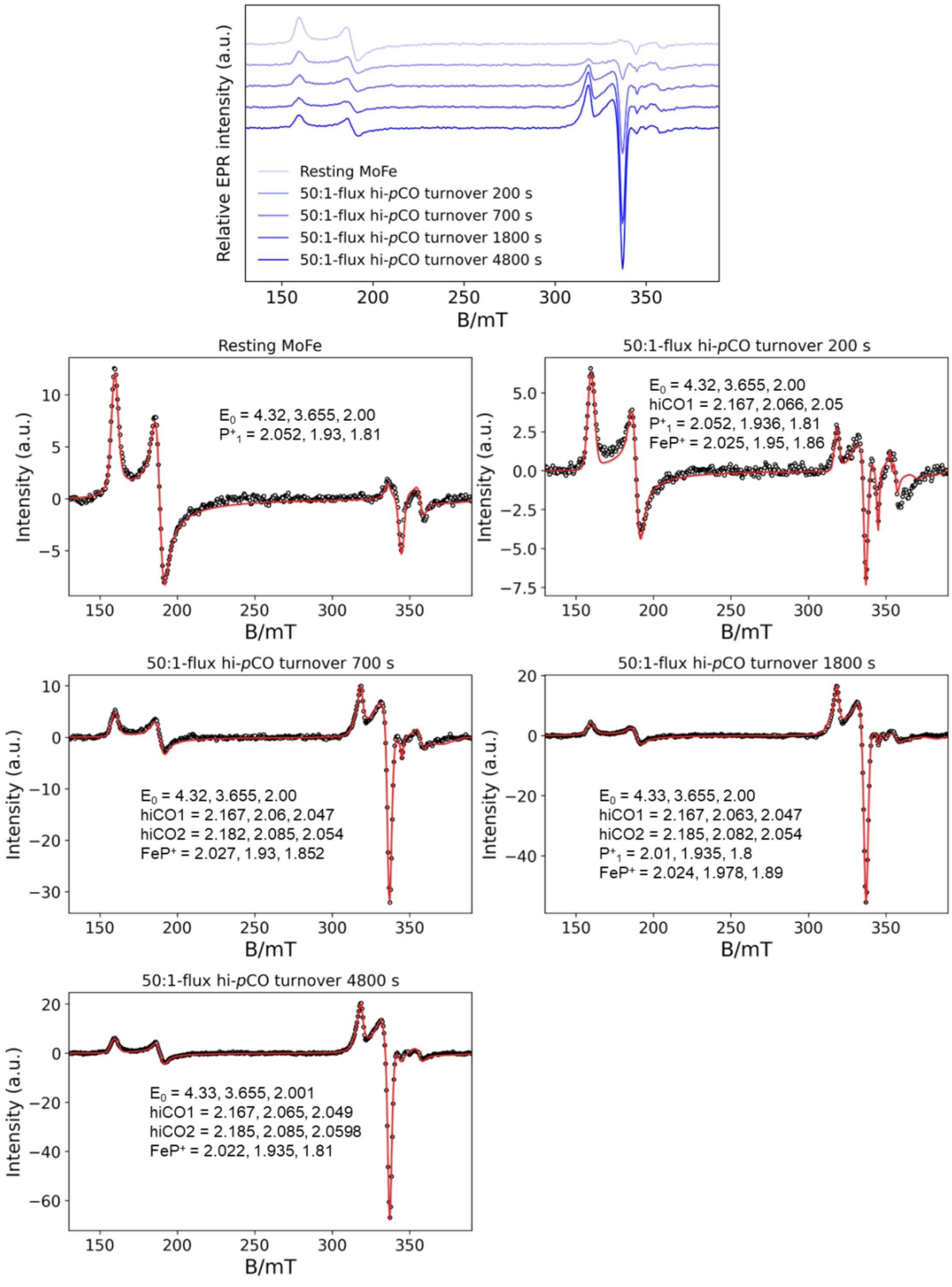

Figure S2: Overlaid EPR spectra of multiple time points with 50:1-flux and hi-*p*CO (top), rest the experimental EPR spectrum (depicted as a black circle) alongside its corresponding fits (illustrated as a red line) in different time points as mentioned above the individual plot, in addition to the resting FeMoco. The resting MoFe overlay plot contains an additional rhombic $S = 1/2$ with resting $S = 3/2$ FeMoco EPR signal is attributed to a background oxidized P-clusters outside our region of interest.

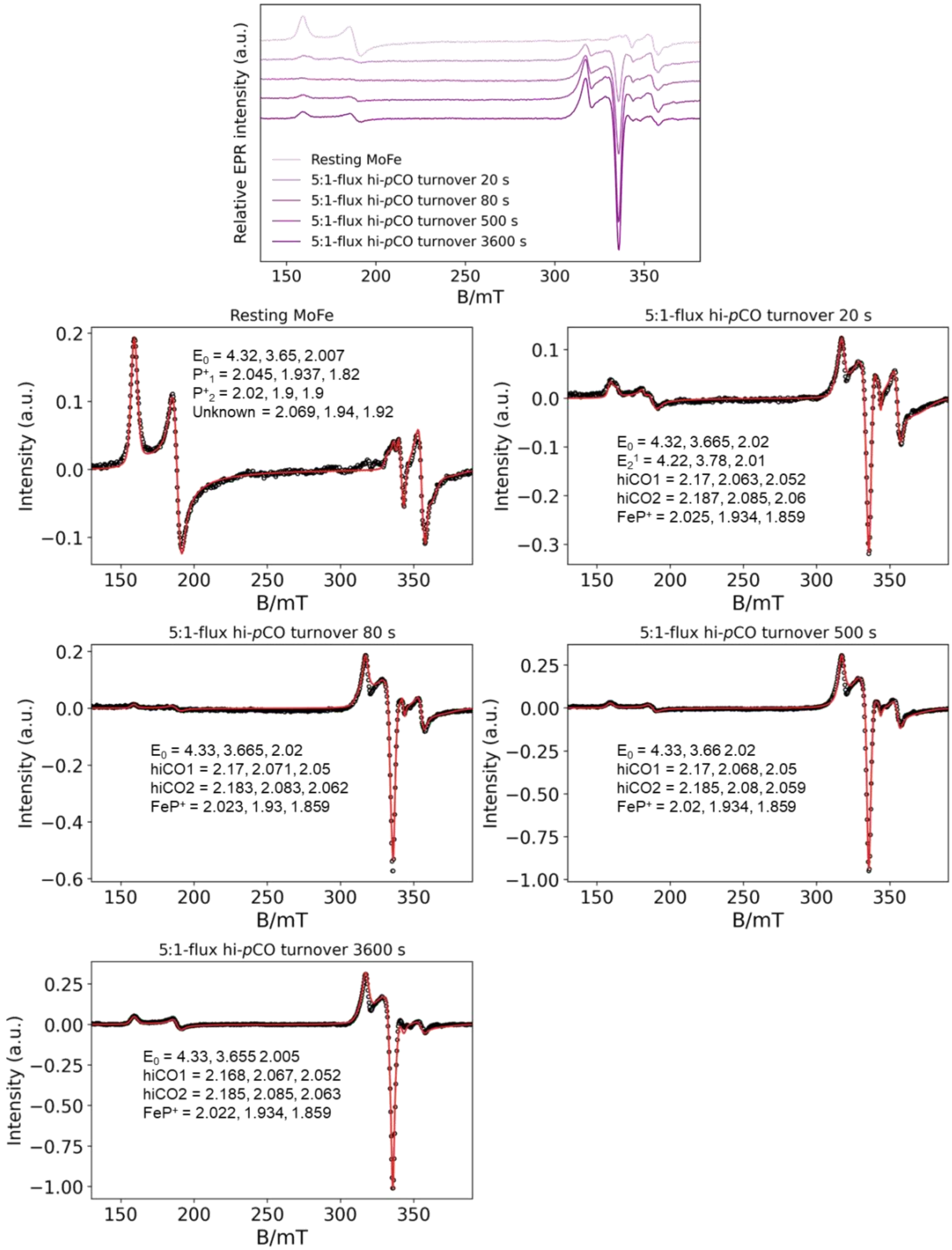

Figure S3: Overlaid EPR spectra of multiple time points with 5:1-flux and hi-*p*CO (top), rest the experimental EPR spectrum (black circle) alongside its corresponding fits (red line) in different time points as mentioned above the individual plot, in addition to the resting FeMoco. The resting MoFe overlay plot contains an additional rhombic $S = 1/2$ with resting $S = 3/2$ FeMoco EPR signal is attributed to a background oxidized P-clusters with some unknown species outside our region of interest.

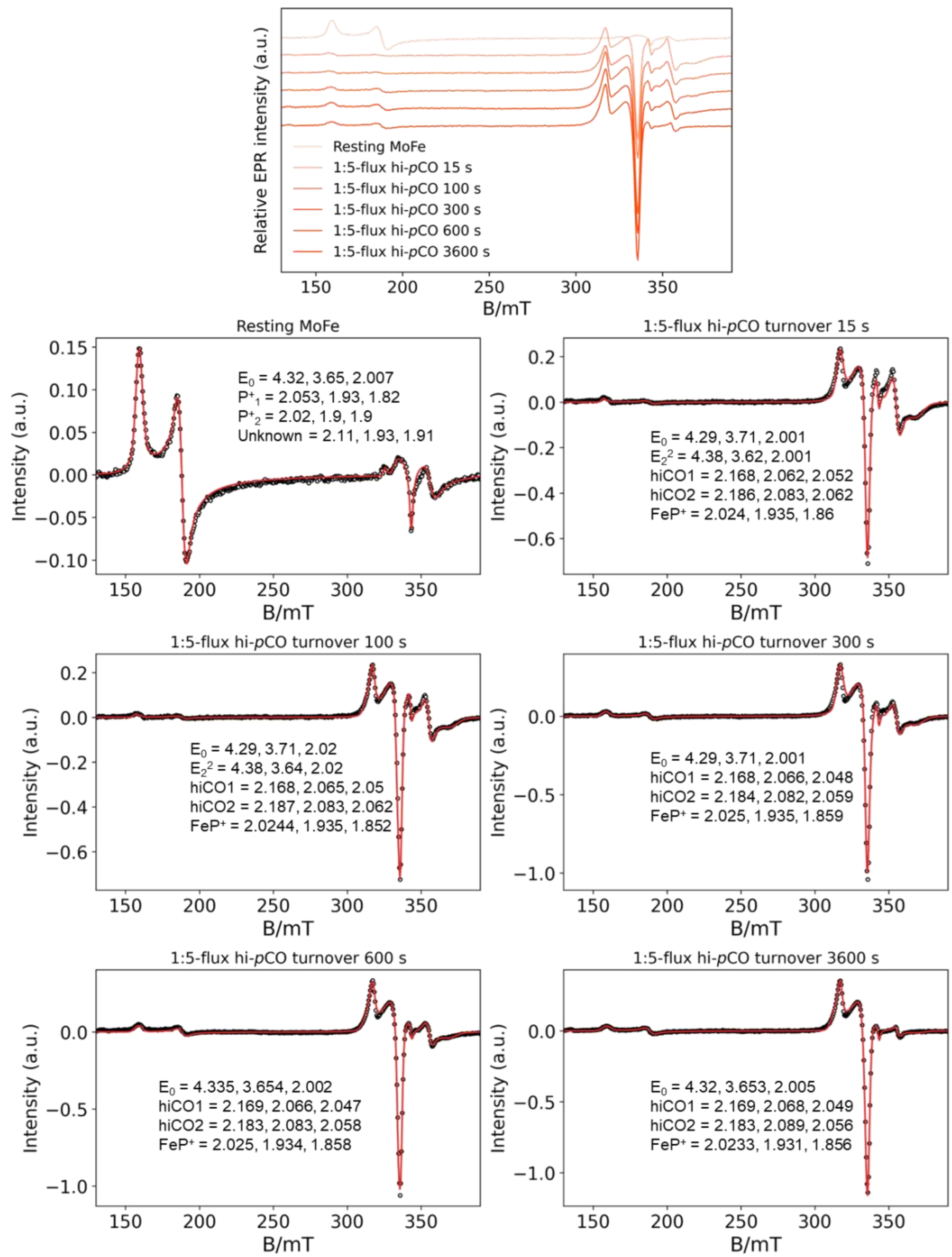

Figure S4: Overlaid EPR spectra of multiple time points with 1:5-flux and hi-*p*CO (top), rest the experimental EPR spectrum (black circle) alongside its corresponding fits (red line) in different time points as mentioned above the individual plot, in addition to the resting FeMoco. The resting MoFe overlay plot contains an additional rhombic $S = 1/2$ with resting $S = 3/2$ FeMoco EPR signal is attributed to a background oxidized P-clusters with some unknown species outside our region of interest.

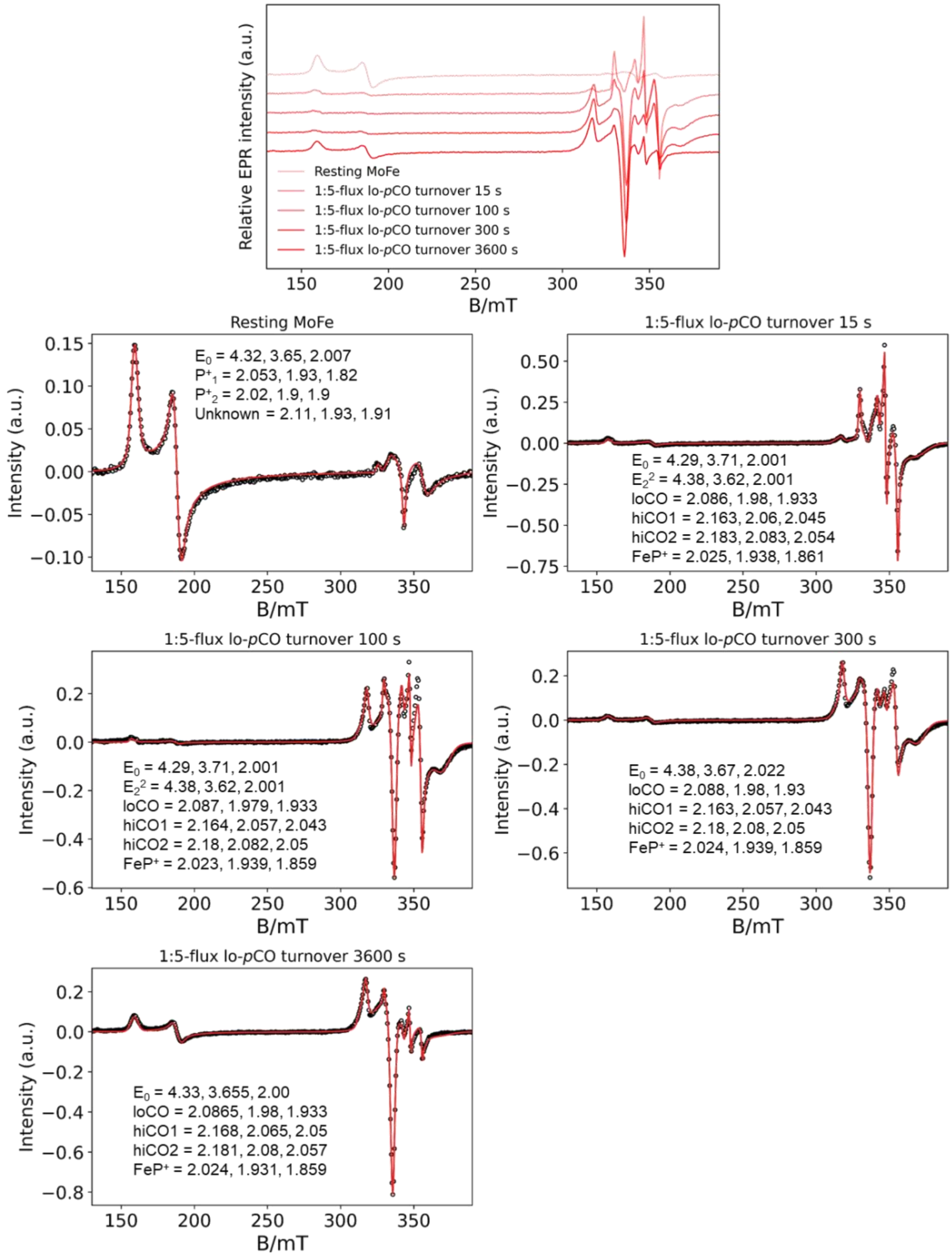

Figure S5: Overlaid EPR spectra of multiple time points with 1:5-flux and lo-*p*CO (top), rest the experimental EPR spectrum (depicted as a black circle) alongside its corresponding fits (illustrated as a red line) in different time points as mentioned above the individual plot, in addition to the resting FeMoco. The resting MoFe overlay plot contains an additional rhombic $S = 1/2$ with resting $S = 3/2$ FeMoco EPR signal is attributed to a background oxidized P-clusters with some unknown species outside our region of interest.

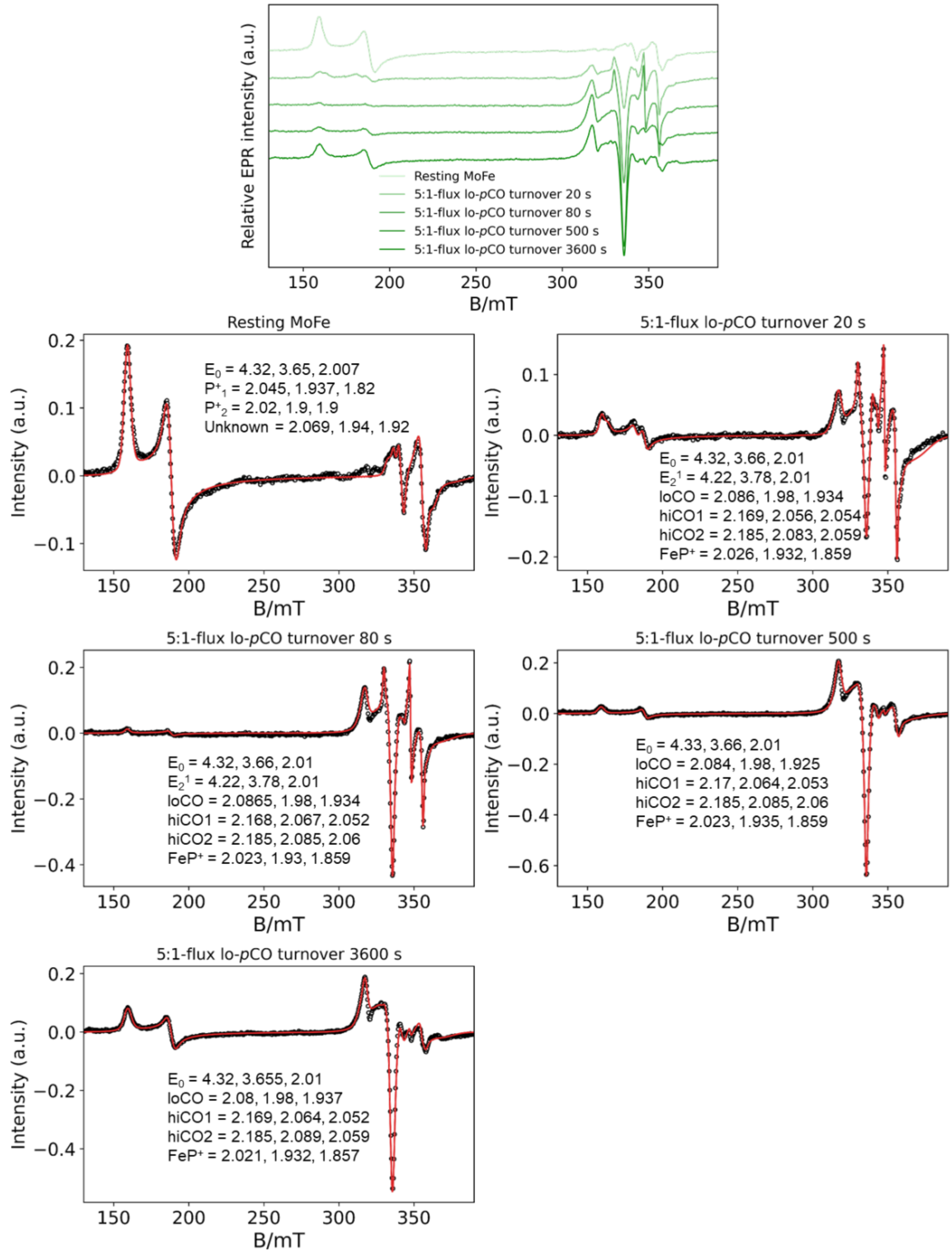

Figure S6: Overlaid EPR spectra of multiple time points with 5:1-flux and lo-*p*CO (top), rest the experimental EPR spectrum (black circle) alongside its corresponding fits (red line) in different time points as mentioned above the individual plot, in addition to the resting FeMoco. The resting MoFe overlay plot contains an additional rhombic $S = 1/2$ with resting $S = 3/2$ FeMoco EPR signal is attributed to a background oxidized P-clusters with some unknown species outside our region of interest.

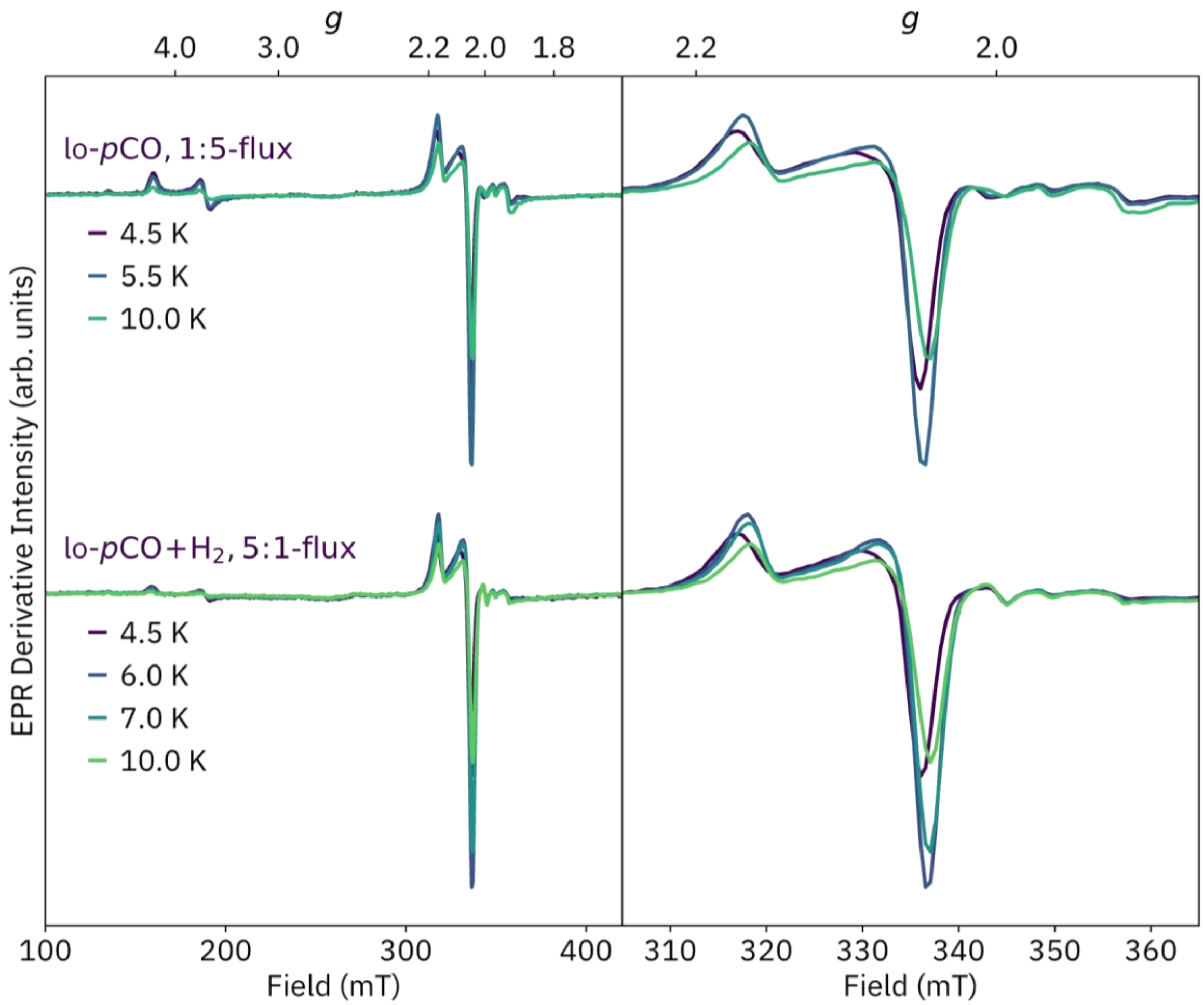

Figure S7: EPR spectra collected at different temperatures for lo-*p*CO and 5:1-flux condition, with and without H_2, showing different relaxation behavior for the two $S = 1/2$ "hiCO" signals. The samples with and without H_2 were freeze-quenched after 300 and 500 s, respectively. Under both reaction conditions, a distinct shift toward lower g-values was observed with increasing temperature (from 4.5 K to 10 K) in the g_1 (~2.17) and g_3 (~2.06) regions. Full spectral simulation revealed that the higher g_1 and g_3 components, predominant at lower temperatures (<5 K), correspond to the hiCO2 state, whereas the lower g_1 and g_3 components, dominant at higher temperatures (>6 K), correspond to the hiCO1 state.

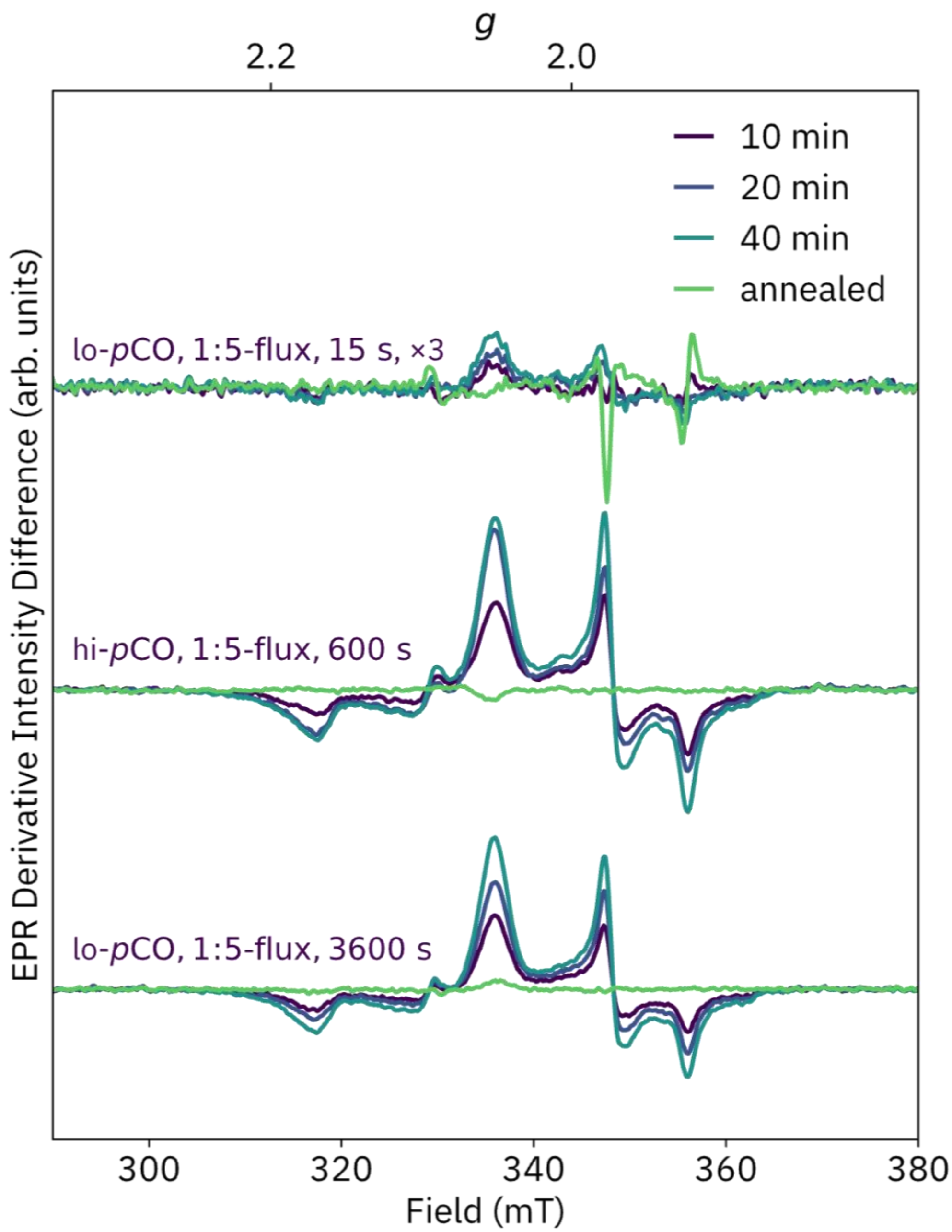

Figure S8: EPR difference spectra from photo-irradiation for 10, 20 and 40 minutes at 10 K, and after subsequent annealing at 77 K. Top: turnover sample at 15 s under low CO partial pressure and a 1:5 flux, showing maximum EPR-loCO accumulation (35%, Table S3), shown with 3x intensity for clarity. Middle: turnover sample at 3600 s under low CO partial pressure and high (1:5) electron flux, showing longer turnover. Bottom: turnover sample at 600 s under high CO partial pressure and high (1:5) electron flux, showing maximum EPR-hiCO accumulation (78%, Table S2).

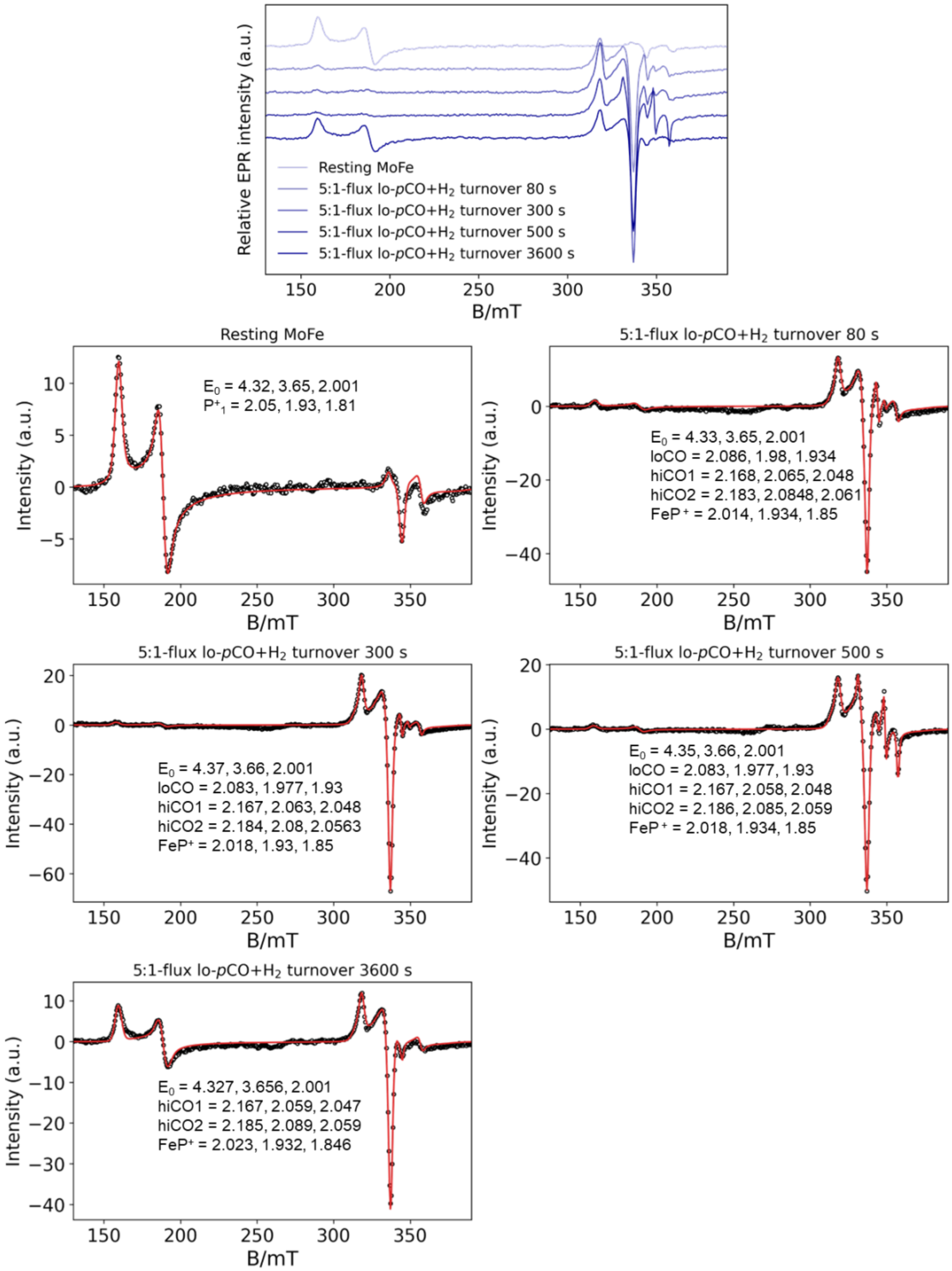

Figure S9: Overlaid EPR spectra of multiple time points with 1:5-flux and lo-*p*CO with 43% H_2 saturation (top), rest the experimental EPR spectrum (black circle) alongside its corresponding fits (red line) in different time points as mentioned above the individual plot, in addition to the resting FeMoco. The resting MoFe overlay plot contains an additional rhombic S = 1/2 with resting S = 3/2 FeMoco EPR signal is attributed to a background oxidized P-clusters outside our region of interest

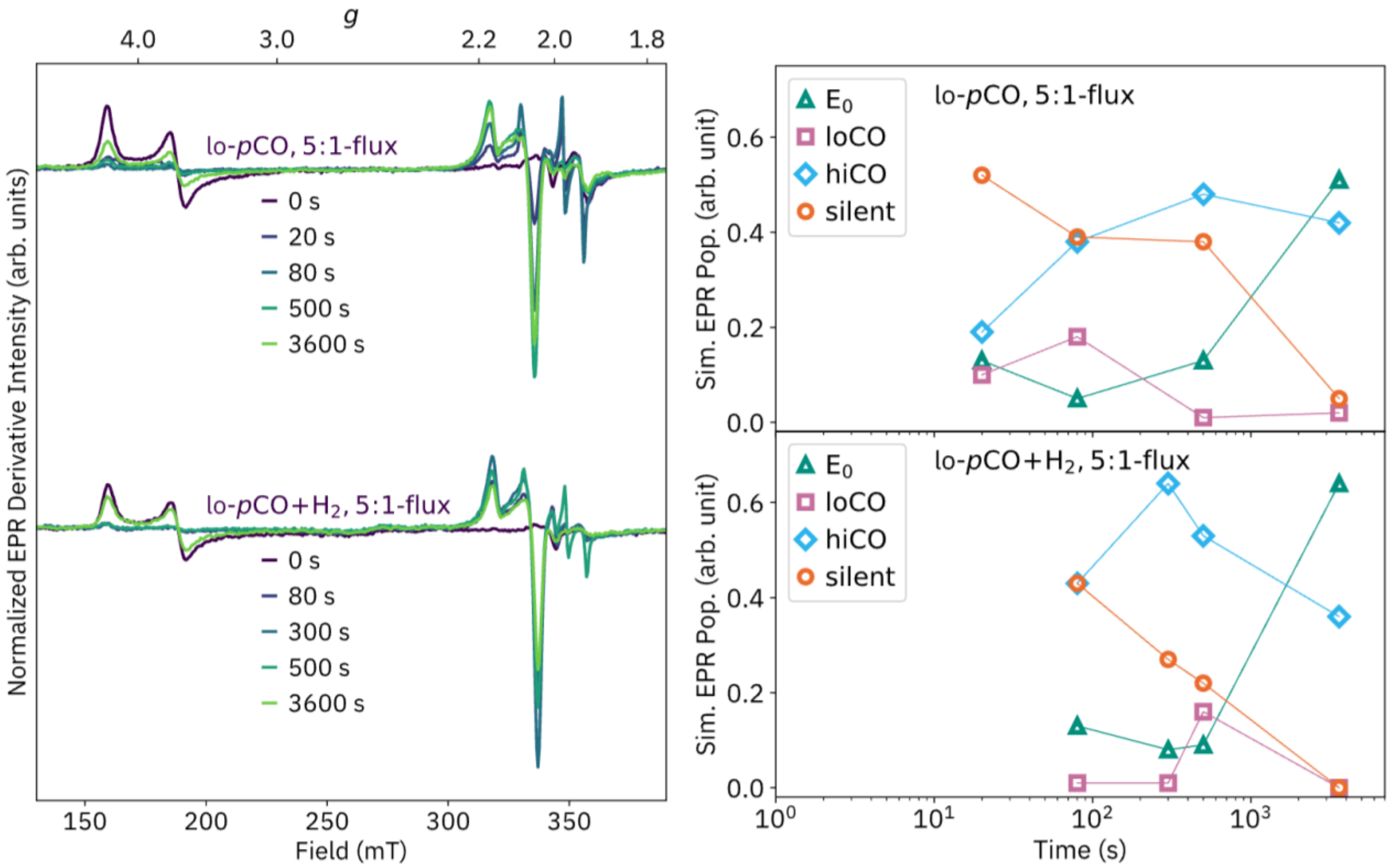

Figure S10: Left: time-dependent EPR spectra for lo-*p*CO pressure and 5:1-flux condition, with (bottom) and without (top) H_2. Right: relative concentration of different intermediates at different time points based on EPR simulation.

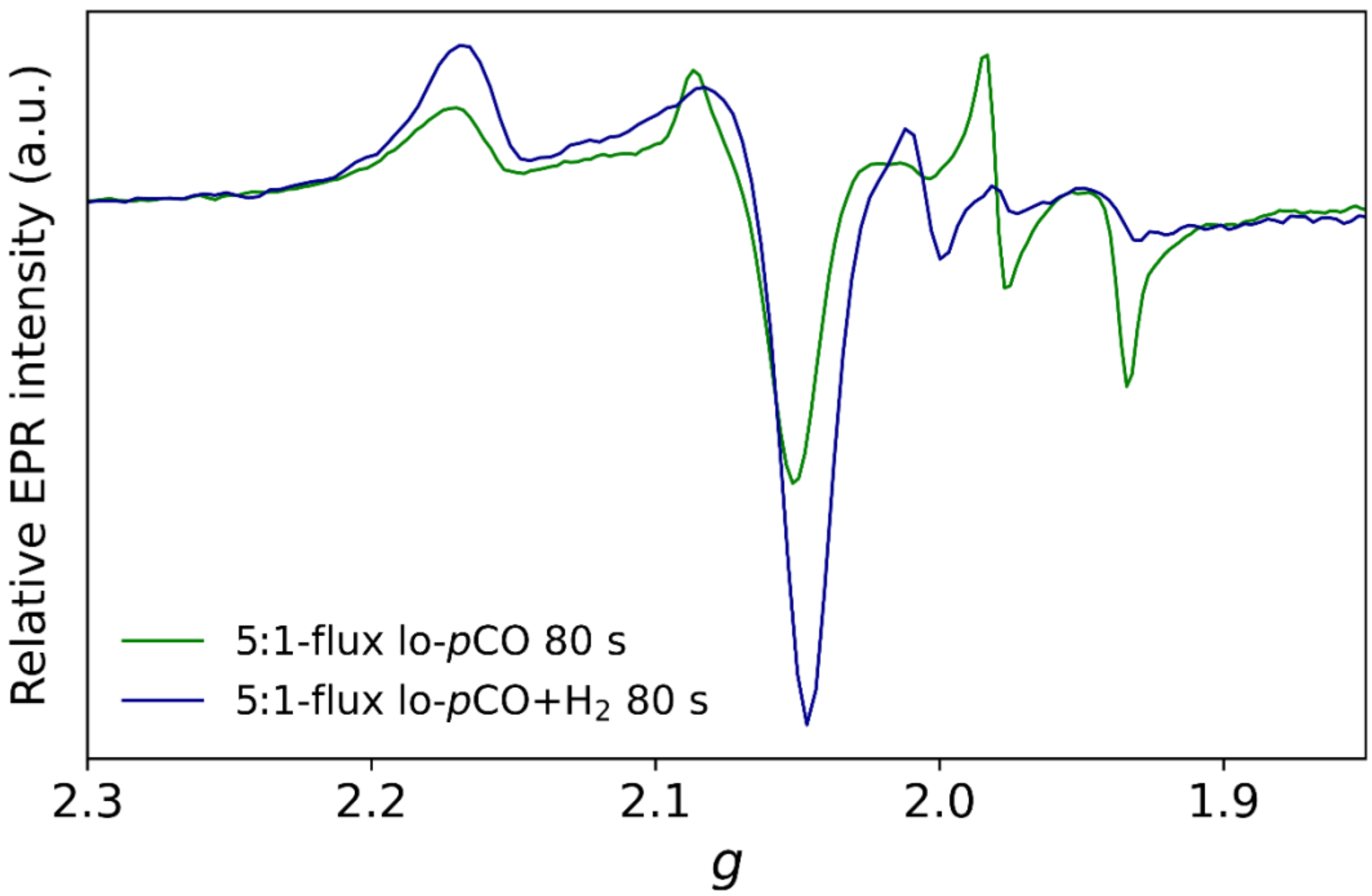

Figure S11: Overlaid spectra for the lo-*p*CO pressure and 5:1-flux condition, with (blue) and without (green) H_2, at the 80 s trapped sample. The relative EPR intensities are normalized with the resting MoFe EPR signal.

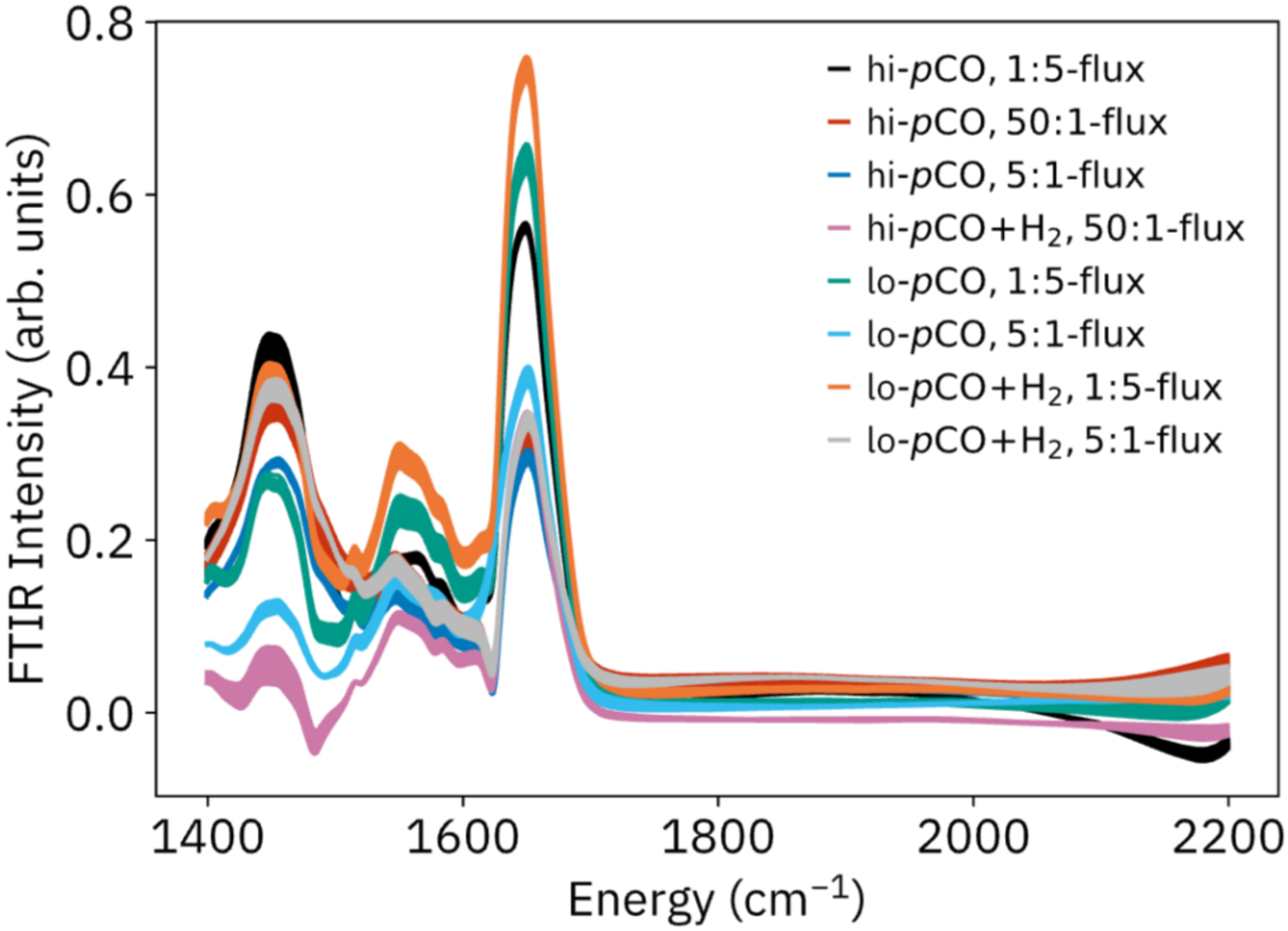

Figure S12: Raw static FTIR datasets for eight different reaction conditions. Variation in the amide bands (1400–1700 cm^{-1}) is a result of variable deuteration of the protein backbone prior to FTIR experiments.

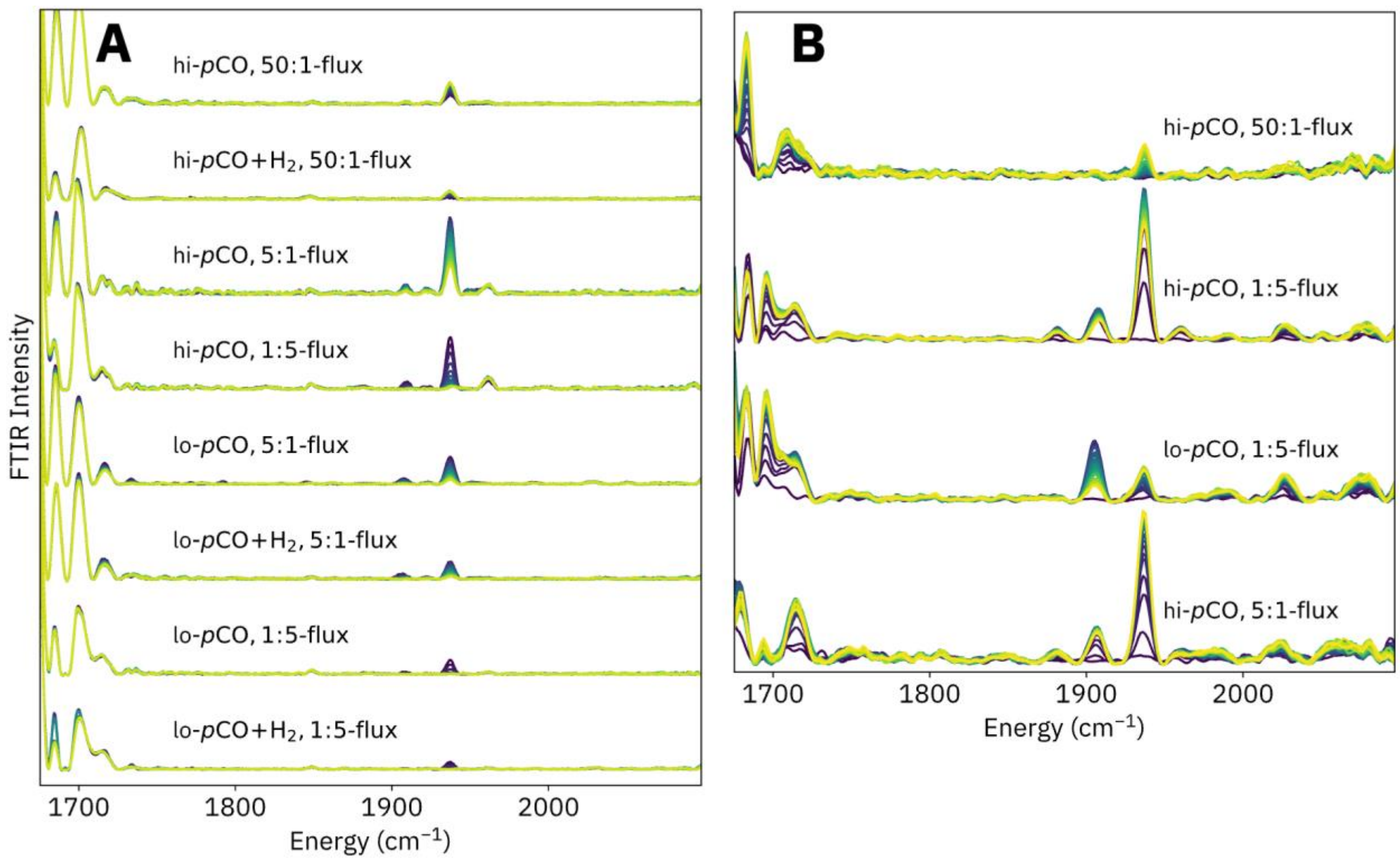

Figure S13: Baseline-subtracted and normalized FTIR spectra for static (A) and stopped-flow (B) datasets, plotted from 1675–2098 cm^{-1}.

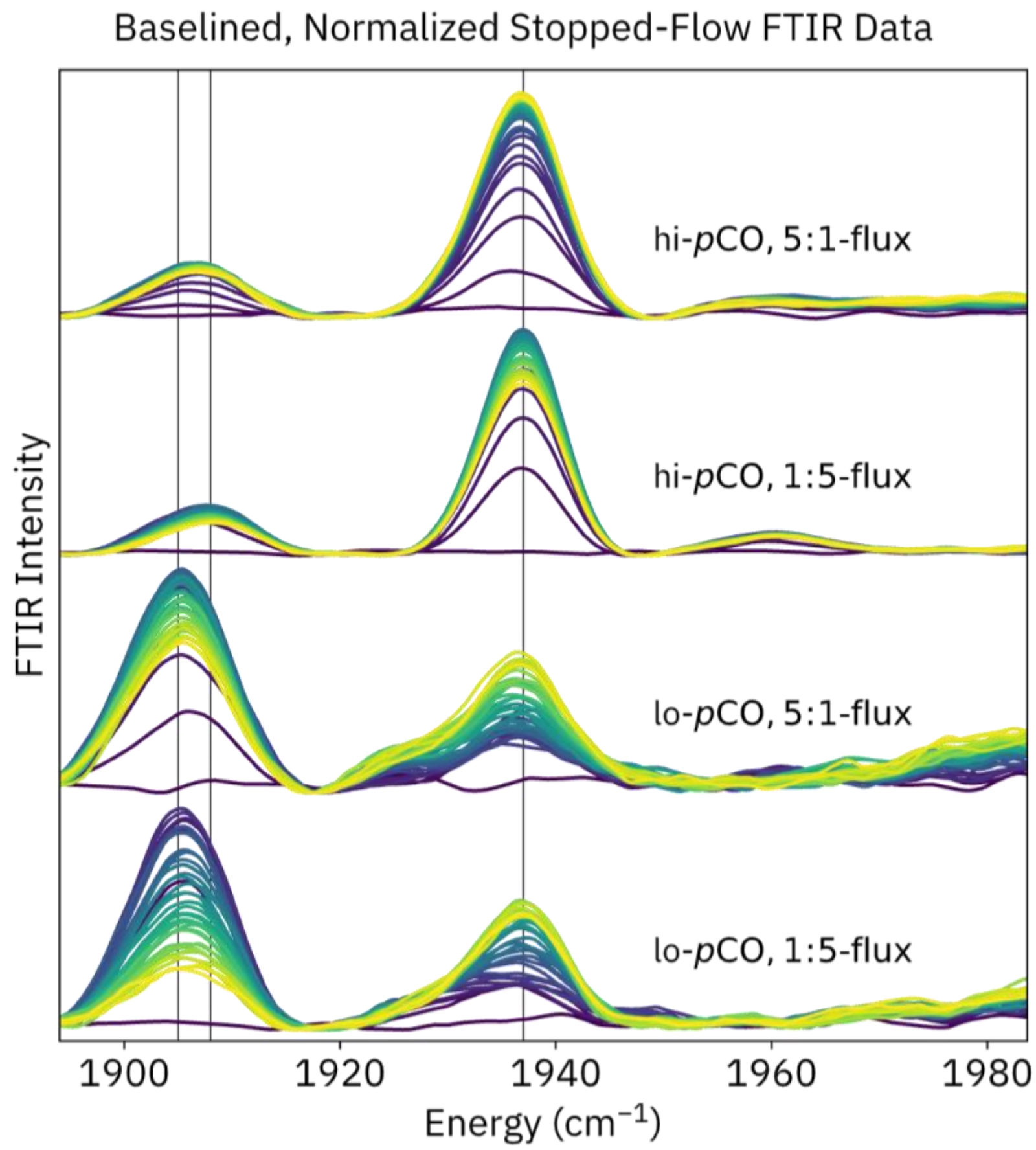

Figure S14: Stopped-flow FTIR spectra, after baseline subtraction and normalization, which are used as input for the NMF analysis. Top: FT-IR overlay of hi-*p*CO and 5:1-flux SF turnover sample data. second: FT-IR overlay of hi-*p*CO and 1:5-flux SF turnover sample data. Third: FT-IR overlay of lo-*p*CO and 5:1-flux SF turnover sample data. Bottom: FT-IR overlay of lo-*p*CO and 1:5-flux SF turnover sample data.

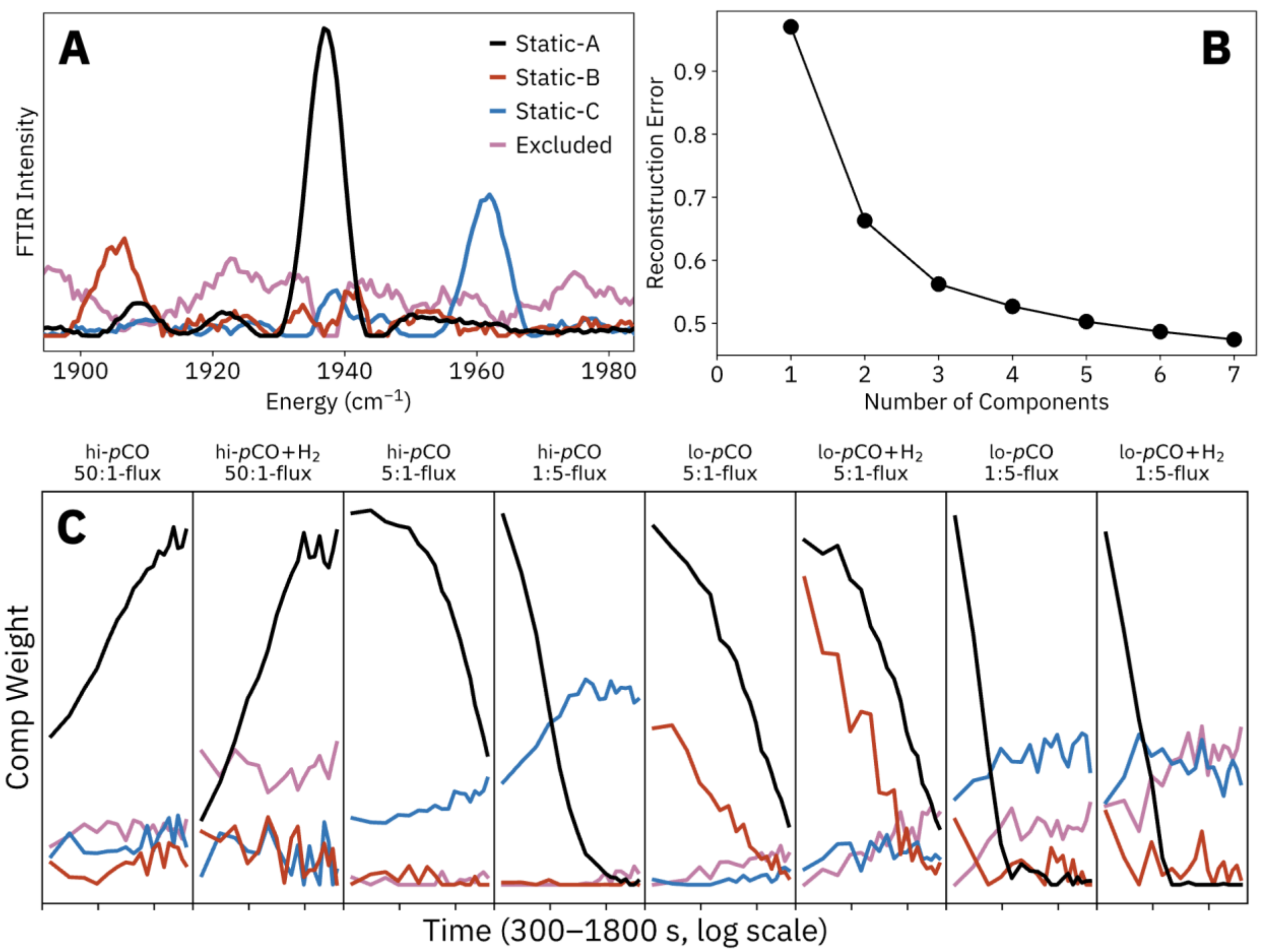

Figure S15: Static NMF analysis, including A) component spectra for a 4-component NMF; B) plot of reconstruction error vs the number of components used for the NMF decomposition; C) trajectories for a 4-component NMF. The excluded component, not shown in the main text, does not have clear peak-like features, apparently spans varying background signal and noise, and is not interpreted chemically. In panel B, although a clear "elbow" is not found at 4 components, 4 components were used for the main NMF analysis because this number of components resulted in chemically interpretable component spectra and trajectories. For the one-component NMF, convergence was not achieved after 10^7 iterations and the optimization was terminated; thus, the plotted value of the reconstruction error with one component is an upper bound.

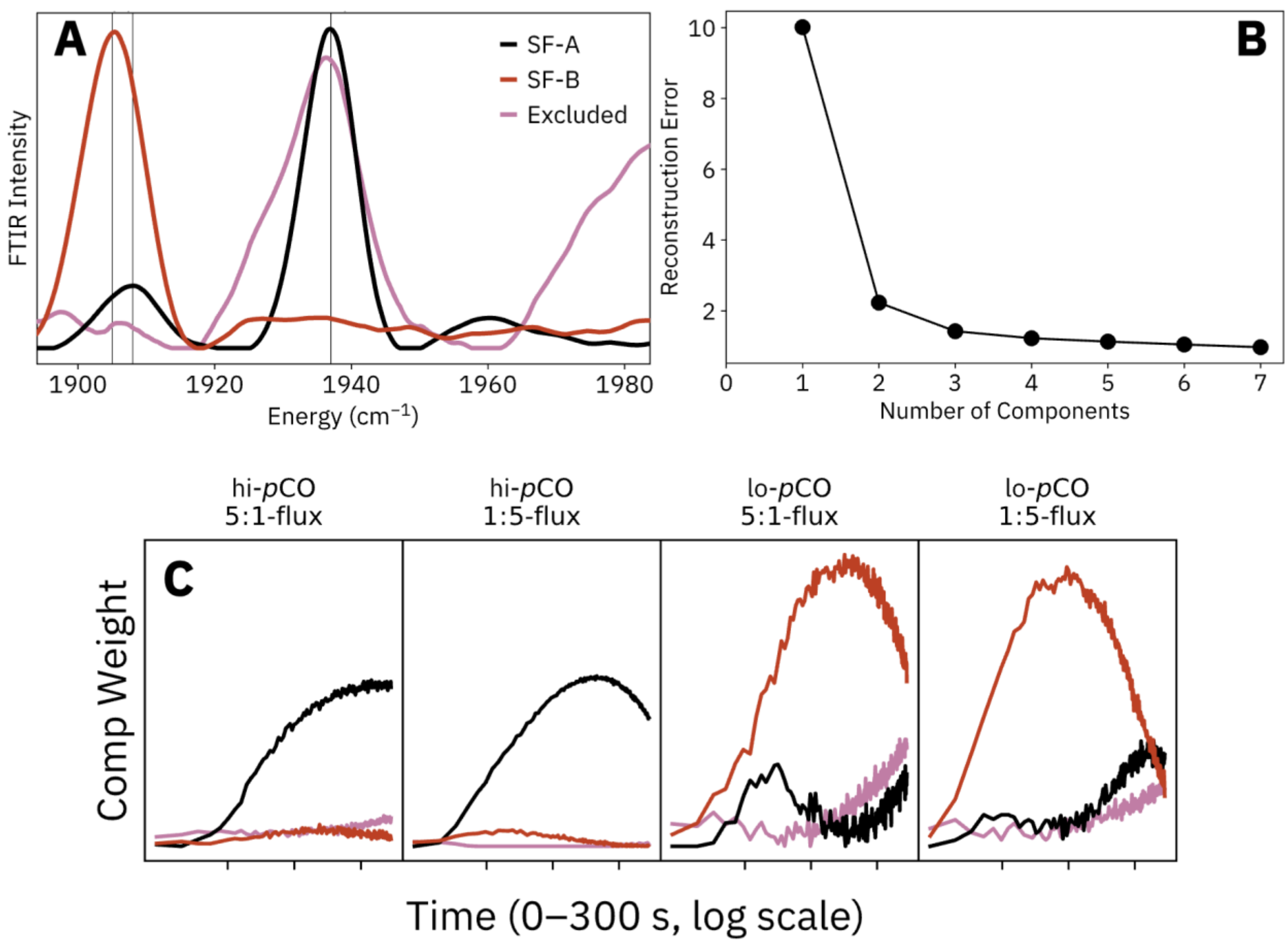

Figure S16: Stopped-flow NMF analysis, including A) component spectra for a 3-component NMF; B) plot of reconstruction error vs the number of components used for the NMF decomposition; C) trajectories for a 3-component NMF. The excluded component, not shown in the main text, contributes to broadening of the 1937 cm^{-1} peak as well as increasing background at higher energy with the time. For the one-component NMF, convergence was not achieved after 10^7 iterations and the optimization was terminated; thus, the plotted value of the reconstruction error with one component is an upper bound.

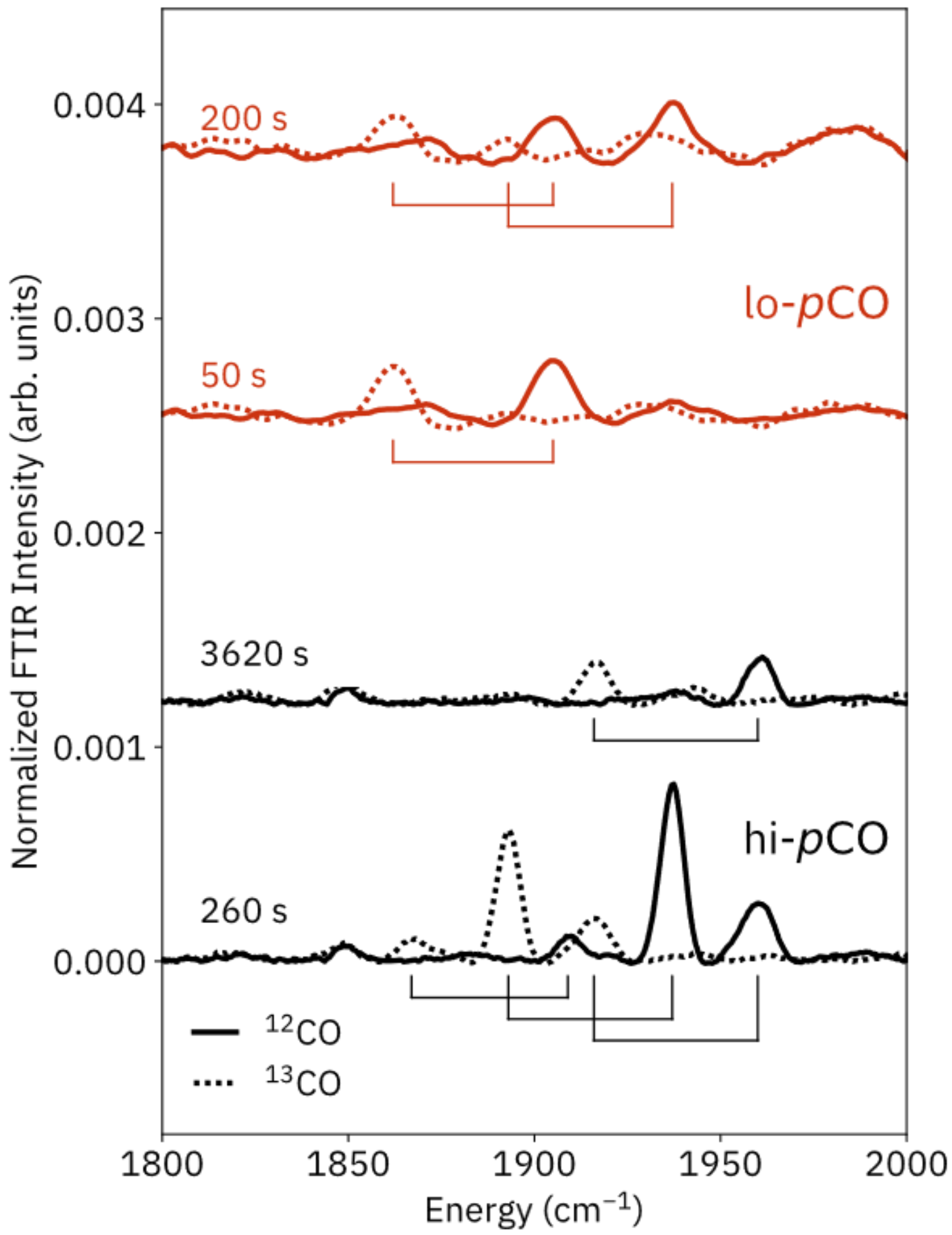

Figure S17. Overlaid FTIR spectra with ^{12}CO (solid lines) and ^{13}CO (dotted lines). Red color: Stopped-flow FTIR data with 1:5-flux and lo-*p*CO condition, Black color: static FTIR data with 1:5-flux and hi-*p*CO reaction condition. Isotope shifts $^{12\rightarrow 13}CO$ of -42 to -44 cm^{-1} are marked below each pair of spectra.

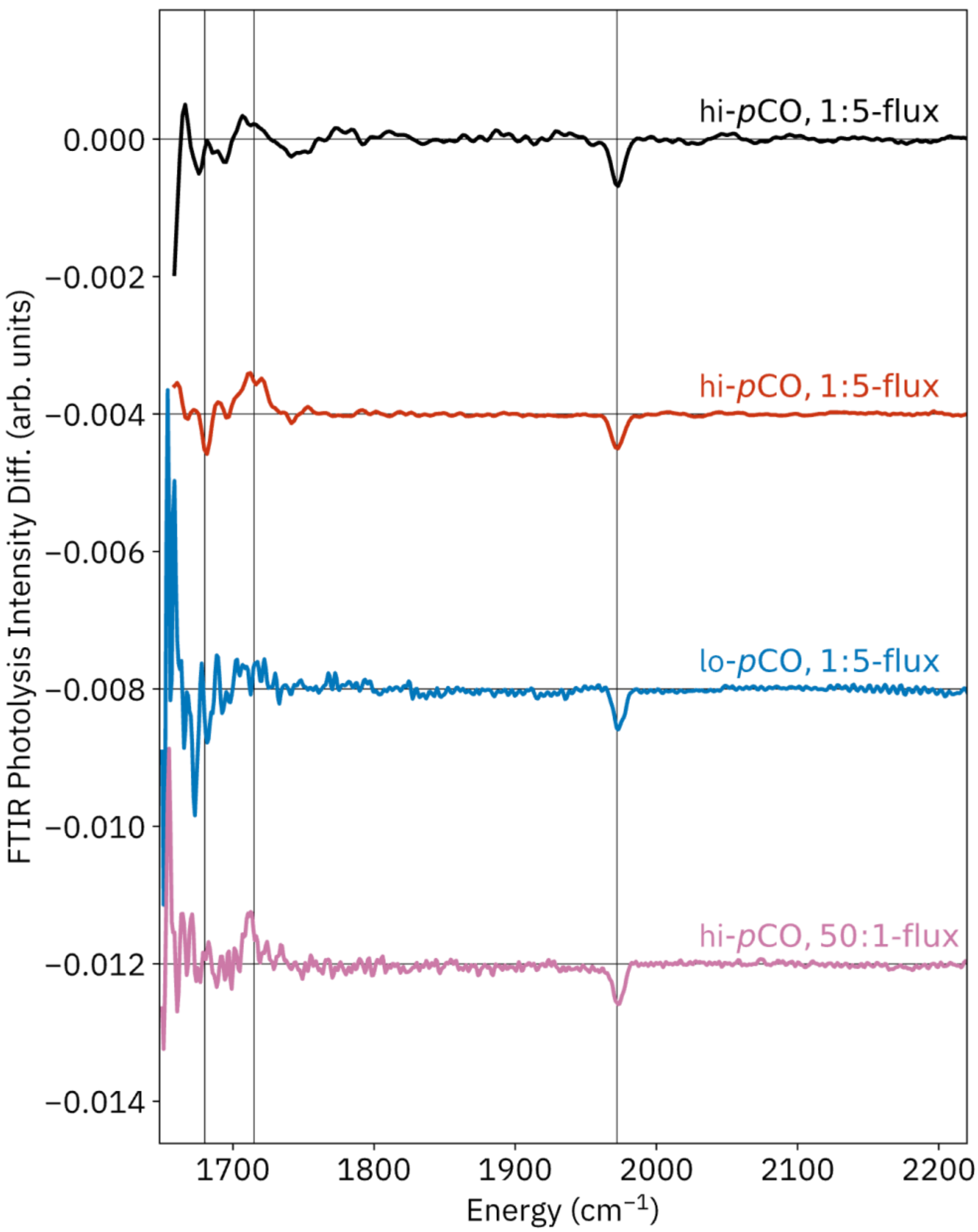

Figure S18: FTIR difference spectra showing the effects of in-situ irradiation with a 24 mW, 468 nm LED at 10 K. From top to bottom: turnover sample at 100 s under hi-*p*CO and 1:5-flux; turnover sample at 600 s under hi-*p*CO and 1:5-flux; turnover sample at 3600 s under lo-*p*CO and 1:5-flux; turnover sample at 3600 s under hi-*p*CO and 50:1-flux. Actual measurement time required further 1800 s addition to the sample quenching time (mentioned earlier). Thin lines are drawn horizontally at the average intensity of the featureless regions of 2000–2150 cm^{-1} for each spectrum and vertically at 1680, 1715, and 1972 cm^{-1}.

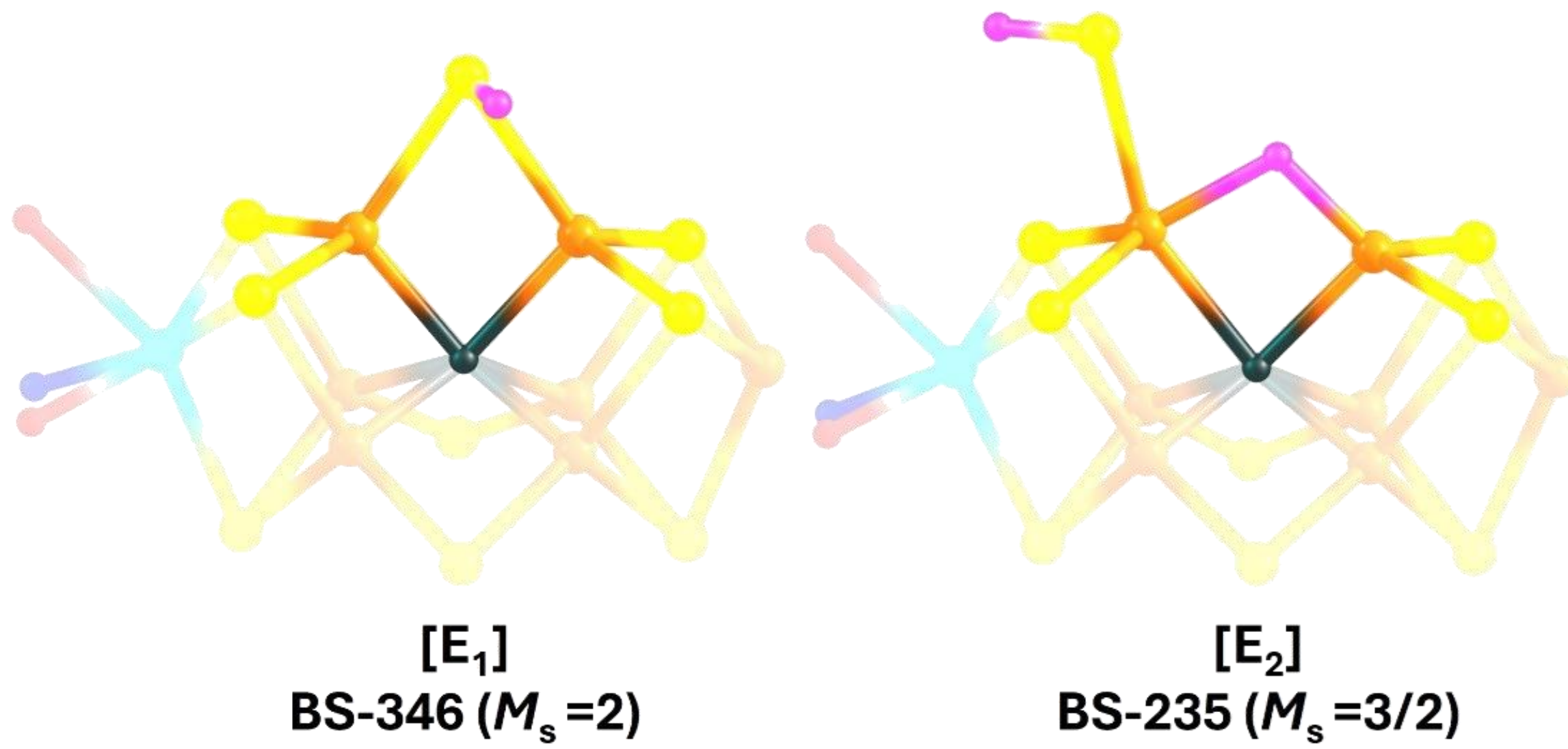

Figure S19: The lowest energy structures for the E_1 (*left*) and E_2(*right*) states of FeMoco, their respective BS-solution labeled below. The primary coordination sphere of the Fe2 and Fe6 sites are highlighted for clarity.

Procedure Followed to Incorporate two Independent Components for EPR-hiCOs: The accumulation of EPR-active subcomponents was quantified for all turnover reactions under varying partial pressures of N_2, CO, and H_2 and across three distinct electron-flux conditions. Concentrations of individual components were determined independently from simulated sub-spectra produced with esimX.SL software. For every individual spectrum to obtain the overall best-fit simulated spectra, in the S = ½ region (the hiCO EPR signal region, g1 ~ 2.17), it was necessary to model two different species (EPR-hiCO1 and hiCO2) using their respective g-values (mentioned in supporting figures S2–S6 and S9) and the best-fit simulated spectra were generated by minimizing the Root Mean Square Error (RMSE) between experimental and simulated data. When simulations were performed using only a single EPR-hiCO component in samples containing more than 10% total EPR-hiCO, the RMSE ratio ($RMSE_{single\text{-}hiCO}/RMSE_{two\text{-}hiCOs}$) increased by more than twofold in most cases. The only condition for which a satisfactory fit was obtained with EPR-hiCO1 alone (~5%) was at the earliest time point (200 s) under the 50:1 electron-flux regime. Therefore, to accurately determine the relative populations of EPR-hiCO1 and EPR-hiCO2 in each CO-turnover sample, their abundances were systematically varied in ± 5% (RMSE ratio > 1.2) increments relative to the initial (t = 0 s) resting MoFe spin concentration, which served as an internal standard. Additionally, we applied the same strategy to obtain the errors in the estimated values for other components (with >6% population) related to FeMoco cluster species. The combinations that yielded the lowest RMSE are listed in the supporting table (Table S2-S4).

Table S1 Quantification of the EPR signals shown in Figure S7. Percentages were calculated using the $S = 3/2$ signal of resting state FeMoco as a reference. The reaction was performed at atmospheric pressure of N_2 in the absence of CO with the indicated electron flux. All the values are corrected by Aasa-Vänngård factor.

[MoFe]:[FeP]	E_0 (%)	E_2^1 (%)	E_2^2 (%)	E_2^3 (%)	$E_{4(2N2H)}$ (%)	$E_{4(4H)}$ (%)	EPR silent (%)
1:0 (resting MoFe)(t = 0s)	100	0	0	0	0	0	0
50:1 (t = 700 s)	47	0	0	0	0	0	53
5:1 (t = 20 s)	4.5	0	2.3	9.0	3.0	1.0	81
1:5 (t = 15 s)	5.1	1.0	5.3	12.8	4.2	1.5	70

Table S2. Quantification of the EPR signals has been made from the simulated spectrum, shown in Figure S8, S9 & S10. Percentages were calculated using the $S = 3/2$ signal of resting state FeMoco as a reference. All the values are corrected by Aasa-Vänngård factor. All the samples were prepared at high partial pressure of CO (0.5 atmosphere). The estimated error limits are tabulated through minimizing the RMSE value for species associated with the FeMoco cluster.

Condition	Component (%)						
([MoFe]:[Fe])	Time (s)	E_0 (±5)	E_2	EPR-hiCO: EPR-hiCO1 (±5)	EPR-hiCO: EPR-hiCO2 (±5)	EPR-hiCO: Total	EPR silent
Resting FeMoco	0	100	0	0	0	0	0
(50:1)	200	38	-	5	0	5	57
	700	36	-	17	14	31	33
	1800	39	-	19	33	52	9
	4800	39	-	42	20	62	0
(5:1)	20	13	6	23	8	31	50
	80	5	-	22	23	45	50
	500	14	-	35	37	72	14
	3600	28	-	39	33	72	0
(1:5)	15	6	-	30	23	53	41
	100	6	-	28	22	50	44
	300	15	-	23	49	72	13
	600	22	-	21	57	78	0
	3600	22	-	32	42	74	4

Table S3. Quantification of the EPR signals from the simulated spectra, shown in Figure S11 & S12. Percentages were calculated using the $S = 3/2$ signal of resting state FeMoco as an internal reference. All the values are corrected by Aasa-Vänngård factor. All the samples were prepared at low partial pressure of CO (0.08 atmosphere). The estimated error limits are tabulated through minimizing the RMSE value for species associated with the FeMoco cluster.

Condition ([MoFe]:[Fe])	Time (s)	E_0 (±5)	E_2	EPR-loCO (±3)	hiCO: EPR-hiCO1(±5)	hiCO: EPR-hiCO2(±5)	hiCO: total	EPR silent
Resting FeMoco	0	100	0	0	0	0	0	0
(1:5)	15	7	2	41	3	4	7	43
	100	4	-	17	18	24	42	37
	300	10	-	5	21	29	50	35
	3600	41	-	8	12	39	51	1
(5:1)	20	13	6	10	10	9	19	52
	80	5	-	18	17	21	38	39
	500	13	-	1	23	25	48	38
	3600	51	-	2	18	24	42	5

Table S4: Quantification of the EPR signals shown in Figure S15. Percentages were calculated using the $S = 3/2$ signal of resting state FeMoco as an internal reference. All the values are corrected by Aasa-Vänngård factor. All the sample were prepared at lo-*p*CO pressure (0.08 atmosphere) with 43% H_2 saturated buffer. The estimated error limits are tabulated through minimizing the RMSE value for species associated with the FeMoco cluster.

Condition	Component (%)							
([MoFe]:[Fe])	Time (s)	E_0 (±5)	E_2	EPR-loCO (±3)	EPR-hiCO: EPR-hiCO1(±5)	EPR-hiCO: EPR-hiCO2(±5)	total	EPR silent
Resting FeMoco	0	100	0	0	0	0	0	0
(5:1)	80	13	-	1	29	14	43	43
	300	8	-	1	37	27	64	27
	500	9	-	16	34	19	53	22
	3600	64	-	0	22	14	36	0

Table S5. Hirshfeld Spin Population Analysis of the E_1 and E_2 states of FeMoco and their CO-bound isomers, with respect to their lowest energy BS-solution (Table S6.)

State	Mo	Fe1	Fe2	Fe3	Fe4	Fe5	Fe6	Fe7
E_1	0.2β	3.2α	2.9α	2.8β	2.9β	2.7α	2.0β	2.6α
$[E_1](CO)_{Fe2}$	0.3β	3.1α	1.8β	2.9β	2.9α	2.6β	2.5α	2.5α
$[E_1](\mu_2CO)$	0.9β	3.1α	2.4β	2.7β	2.7β	2.7α	1.5α	2.7α
$[E_1](CO)_{Fe6}$	0.3β	3.2α	2.9α	2.9β	2.9β	2.5α	1.4β	2.4α
E_2	0.3β	3.2α	2.7β	2.8β	3.0α	2.7β	2.6α	2.5α
$[E_2](CO)_{Fe2}$	0.2β	3.1β	3.0α	2.9α	2.9β	2.8α	0.9α	2.3β
$[E_2](\mu_2CO)$ (BS-235)	0.1α	3.2α	2.4β	2.8β	3.0α	2.6β	0.7β	2.6α
$[E_2](\mu_2CO)$ (BS-147)	0.3β	3.2β	2.8α	2.9α	3.0β	2.8α	1.9α	2.5β
$[E_2](CO)_{Fe6}$	0.0β	3.1α	2.4β	2.8β	3.0α	2.7β	0.2β	2.7α
$[E_2](\mu_2CO)(CO)_{Fe2}$	0.3α	3.2α	1.6β	3.0α	3.0β	2.7α	1.5β	2.6β
$[E_2](\mu_2CO)(CO)_{Fe6}$	0.1α	3.1α	2.0β	2.8β	3.0α	2.8β	0.7β	2.5α

Table S6. The relative energies (kcal mol^{-1}) of the select BS-solutions for the E_1 and E_2 states of FeMoco and their CO-bound isomers. The 'X' indicates a BS-solution that didn't converge for a selected structure.

State	BS-235	BS-247	BS-346	BS-147
E_1 (M_s=2)	1.8	3.4	0.0	8.8
$[E_1](CO)_{Fe2}$ (M_s=2)	0.0	1.3	3.1	3.7
$[E_1](\mu_2CO)$ (M_s=1)	0.3	1.5	0.0	9.4
$[E_1](CO)_{Fe6}$ (M_s=2)	10.0	12.6	0.0	23.3
E_2 (M_s=3/2)	0.0	0.8	1.6	7.7
$[E_2](CO)_{Fe2}$ (M_s=1/2)	19.9	21.5	1.7	0.0
$[E_2](\mu_2CO)$ (M_s=1/2)	0.0	1.0	7.6	0.8
$[E_2](CO)_{Fe6}$ (M_s=1/2)	0.0	25.2	5.3	X
$[E_2](\mu_2CO)(CO)_{Fe2}$ (M_s=1/2)	17.2	0.0	31.5	13.5
$[E_2](\mu_2CO)(CO)_{Fe6}$ (M_s=1/2)	0.0	9.8	10.2	16.0
